# Quality of life in patients with pan-cancer undergoing concurrent chemoradiotherapy: a bibliometric analysis (1995-2024)

**DOI:** 10.3389/fonc.2025.1572725

**Published:** 2025-08-12

**Authors:** Ao Shen, Pan Fan, Dingrong Fan, Yidi Wang, Kailin Tang, Ying Cai, Hengyu Zhou

**Affiliations:** ^1^ School of Nursing, Chongqing Medical University, Chongqing, China; ^2^ Department of Oncology, the Second Affiliated Hospital of Chongqing Medical University, Chongqing, China; ^3^ Department of Pediatrics, the Second Affiliated Hospital of Chongqing Medical University, Chongqing, China; ^4^ Department of breast and thyroid surgery, the First Affiliated Hospital of Chongqing Medical University, Chongqing, China; ^5^ Department of Gastroenterology, the First Affiliated Hospital of Chongqing Medical University, Chongqing, China

**Keywords:** bibliometric, chemoradiotherapy, quality of life, concurrent chemoradiotherapy (CCRT), oncology

## Abstract

**Background:**

Chemoradiotherapy is a therapeutic approach that prolongs survival but may simultaneously negatively affect the quality of life (QOL) of cancer patients. Current research on quality of life (QOL) in pan-cancer patients undergoing concurrent chemoradiotherapy (CCRT) lacks systematic integration of bibliometric findings with clinical symptom data.

**Methods:**

We retrieved 2762 articles from the Web of Science Core Collections. R-bibliometrix, VOSviewer, and CiteSpace were employed to conduct quantitative analysis and visualize research trends and factors influencing QOL. Complementarily, a cross-sectional study of 117 cervical cancer patients assessed symptom prevalence via CTCAE v5.0, with symptom clusters identified.

**Results:**

The included articles were published between 1995 and 2024. The results revealed that the United States and China had the largest number of publications worldwide. Van Berge Henegouwen was the most productive author. The institution leading in this field was the University of Toronto. The International Journal of Radiation Oncology - Biology – Physics was the most productive journal. In addition, keywords with high burst strengths in recent years were ‘open label’, ‘predictor’, and ‘preoperative chemoradiotherapy’. Tree-ring map of terms related to QOL was visualized and multiple clusters were found, respectively named as “malnutrition”, “watch and wait”, and so on. Clusters analyses of specific cancers were performed to reveal these unique differences. Finally, among cervical cancer patients, decreased appetite (79.5%), diarrhea (65.8%), and altered taste (59.0%) were the most prevalent symptoms, with three symptom clusters identified.

**Conclusion:**

More attention was paid to long-term outcome and patient experience during treatment. Through pan-cancer research and in-depth analysis of specific cancers, we have identified various factors affecting QOL in patients undergoing chemoradiotherapy, including treatment methods, treatment-induced symptoms, psychological factors and so on, enabling us to tailor more personalized treatment plans that improve their overall well-being and enhance QOL during and after treatment.

## Introduction

Cancer is a formidable public health challenge globally, which stands as one of the leading causes of death in numerous countries (1). Data from recent estimates of global mortality shows that three out of ten people die of cancer (2). With economic development and population aging, the incidence of cancer is predicted to reach 35 million by 2050 (2), potentially giving rise to huge problems and burdens (3).

Concurrent chemoradiotherapy involves the administration of chemotherapy drugs through oral or intravenous solutions concurrently with radiotherapy (4), reducing the risk of death by 30% to 50% and resulting in a growing population of cancer survivors (5). As an alternative or prior to surgery, it has been widely used and has become a standard treatment for several cancers, like head and neck cancer (6), cervical cancer (5) and rectal cancer (7). As survival rates continuously improve, and treatment courses lengthen, cancer is regarded as a special type of chronic illness for survivors (8). However, the negative impacts of therapeutic approaches on patients are common and should not be overlooked. Both radiotherapy and chemotherapy cause damage to normal tissues and organs to a certain extent, leading to a series of adverse events, such as nausea, vomit, hair loss, fatigue, and decreased immune function (9). The adverse symptoms of chemoradiotherapy may be more severe than those of radiotherapy or chemotherapy alone. Crucially, these treatment-induced symptoms are intrinsically linked to and often directly responsible for diminished quality of life (QOL) in survivors, contributing to unpleasant experiences and functional limitations.

Current health studies on cancer patients mostly employ overall survival or progression-free survival as endpoints (10). Since these data do not fully reflect the overall health status of patients, quality of life (QOL) has become an important indicator (11) of an individual’s survival and health status (12), including physical, psychological, social, and other aspects (13). And it is frequently used interchangeably with health related quality of life in academic literature (14). Consequently, QOL assessments play a pivotal role in cancer treatment. However, the QOL scales possess certain limitations in assessing the quality of life of cancer patients. On the one hand, these scales typically concentrate on several restricted dimensions and vary in their areas of emphasis. Therefore, a single measurement instrument is incapable of comprehensively capturing the multifaceted factors that impact patients’ quality of life during the treatment process (15). For instance, issues related to social and emotional support may be overlooked. On the other hand, these scales have certain limitations in interpreting patients’ subjective feelings and experiences, potentially neglecting the nuanced yet crucial disparities within their illness experiences (16).

Comprehensive analyses of articles in a field that has developed over time are challenging. Bibliometric analyses provide researchers with valuable reference information and can guide their research directions through quantitative evaluations and overviews of the literature characteristics, such as authors, countries, institutions, and keywords (17). The existing academic literature pertaining to the QOL of cancer patients predominantly emphasizes distinct types of tumors, encompassing gastric cancer, gynecological tumors and neoplasms of the head and neck. So, there is still an absence of research that comprehensively examines the QOL of patients undergoing concurrent radiotherapy treatment from a pan-cancer perspective. We aimed to conduct pan-cancer research through bibliometrics methods, exploring common factors that impact quality of life of cancer patients and considering the unique characteristics of specific tumors, with the aim of providing comprehensive and targeted guidance for clinical treatment and care to improve patients’ QOL.

## Methods

### Data source and search strategy

This study reviewed articles utilizing the Web of Science (WOS), which is a comprehensive database containing more than 12,000 high-quality journals and citation records that is widely used by academics (18). The WOS prevails over other databases, such as SCOPUS, PUBMED, and MEDLINE (19). So to ensure the high quality and comprehensiveness of the literature, we chose WOSCC that encompasses both SCIE (Science Citation Index Expanded) and SSCI (Social Science Citation Index) as the data source and searched on 1 July, 2024. MESH (Medical Subject Headings) lists topics specially prepared for the medical literature and covers a wide range of medical-related concepts, helping to improve the accuracy and efficiency of retrieval. The search strategy which mainly included MESH term ‘cancer’, ‘chemoradiotherapy’ and ‘quality of life’ was presented in the **Supplementary Information** (**Supplementary Table S1**). To further compare the change of research focus in different cancer types, we conducted another survey about head and neck cancer, rectal cancer, anal cancer, cervical cancer, bladder cancer and glioblastoma. Search strategy and article numbers were shown in **Supplementary Table S2**.

### Inclusion criteria

Only original articles and reviews written in English were included in the analysis. For peer review, as well as content completeness, we excluded types such as meeting abstract, editorial material, proceeding paper, early access, letter, correction, and book chapter. The retrieved papers, consisting of complete records and cited references, were exported in plain text for further analysis.

### Visualisation and statistical analysis

The study used R Package Bibliometrix 4.3.2 (20), VOSviewer 1.6.19 (21), and CiteSpace 6.1.R6 (22). R-Bibliometrix, a bibliometric analysis package based on the R language, is used to calculate the frequency of keywords, collaborations between countries, and scientific publications by authors, institutions, countries, and journals. Moreover, a three-field plot of author keywords was visualized. VOSviewer with an embedded clustering algorithm (23) was employed to extract key information and high-frequency fields and explore the cooperation network between authors and institutions. CiteSpace is frequently used for bibliometric analyses. This software has attracted widespread attention due to its ability to visualize functions and process data. Thus, this study used it to conduct a burst analysis of keywords and citations and generate keyword clusters. In addition, we visualized international collaborations between countries through the online bibliometrics website (https://bibliometric.com/).

### Chart interpretation

CiteSpace and VOSviewer possess two basic components: nodes and links. A node represents a particular term, such as a country, author, or keyword. The size of a node corresponds to the co-occurrence frequency of the term. These links exhibit similar characteristics. In cluster analyses, nodes that are clustered together have the same color. Different clusters represent different topics in the field. If clusters incorporate time, the color shows research trends. CiteSpace can detect keyword and citation bursts. This function was applied to identify cited references and keywords with strong citation bursts during a certain period.

### Clinical data collection and analysis

A cross-sectional survey was conducted at Chongqing Medical University Second Affiliated Hospital from October 2023 to July 2024 to quantify symptom prevalence in cervical cancer patients undergoing CCRT. The inclusion criteria were as follows: (1) age > 18 years; (2) histopathologically confirmed cervical cancer meeting the criteria for concurrent chemoradiotherapy; and (3) voluntary participation in the study. Exclusion criteria comprised: (1) individuals with communication impairments hindering study completion; (2) those diagnosed with malignancies at other anatomical sites; and (3) participants with intellectual disabilities preventing them from completing the survey. The study protocol was approved by the Ethics Committee of the Second Affiliated Hospital of Chongqing Medical University (Approval No. 2400092595). Symptoms were graded using the Common Terminology Criteria for Adverse Events (CTCAE) v5.0 selected for its standardized assessment of treatment-related toxicities and wide acceptance in oncology clinical trials, covering domains such as nausea, fatigue, anemia, diarrhea, and urinary dysfunction. Symptom severity was categorized as Grade 1 (mild) to Grade 4 (life-threatening).

Data analysis was performed using R language (v4.3.1). Symptom incidence was calculated as the proportion of patients reporting each symptom (Grades ≥1) relative to the total sample, with high-frequency symptoms defined as those occurring in ≥ 20% of cases. To identify clusters of high-frequency symptom, the Louvain algorithm (implemented via the igraph package) was applied to detect clusters.

## Results

### Main information

**Figure 1:**
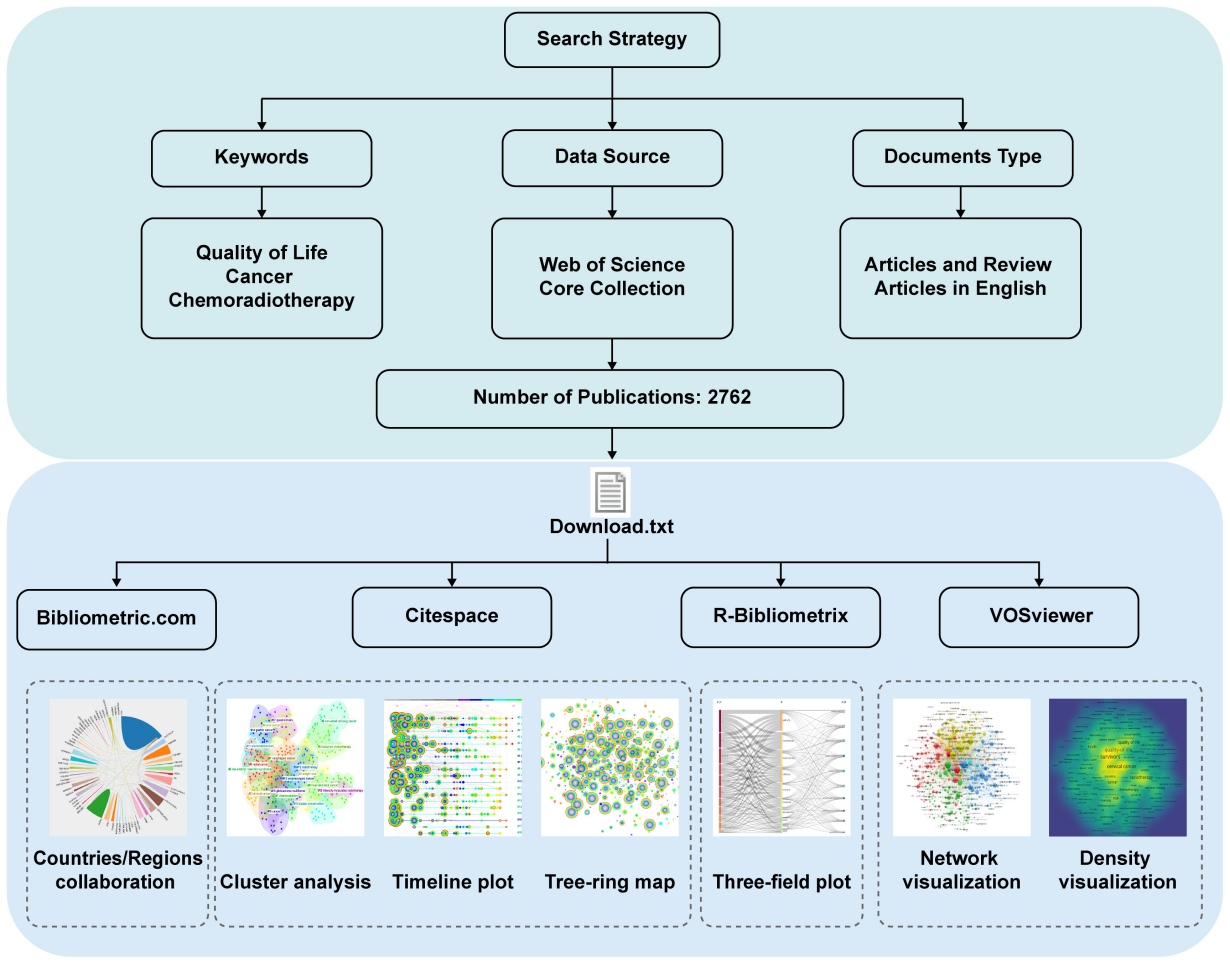
Flow-chart of the study.

As depicted in the flowchart (**Figure 1**), we retrieved 2,932 articles related to the QOL of patients with cancer undergoing concurrent chemoradiotherapy between 1995 and 2024, and 2,762 articles were included (**Supplementary Figure S1A**). These articles were published in 557 journals, contained 67,191 references and involved 3,871 authors. Among the publications, 2,184 (79.1%) were original articles and 578 (20.9%) were reviews. The ratio of the two was nearly 5:1. Furthermore, 3,871 author keywords were extracted by R-Bibliometrix. The average number of citations per article was 33.1.

### Annual scientific production

Of the 2762 articles, 113 (4.1%) were published during the period from 1995 to 2004, 706 (25.6%) from 2005 to 2014, and 1943 (70.3%) from 2015 to 2024. The number of articles published annually has grown steadily since 1995 (**Supplementary Figure S1B**). Before 2017, fewer than 200 articles were published. In 2018, this number reached 207 and peaked at 266 in 2022.

### Contributions by country/region

**Figure 2:**
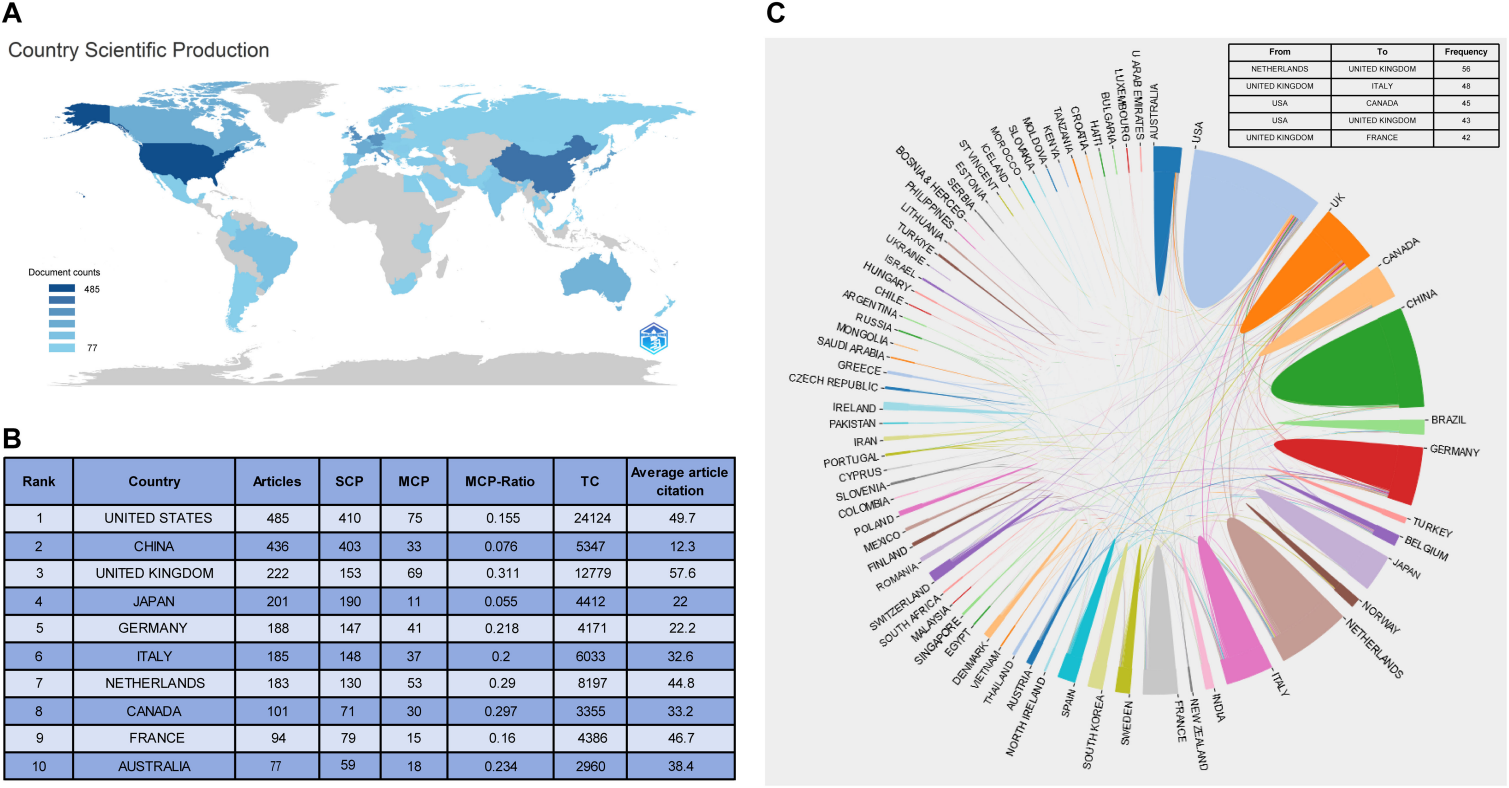
Analysis of countries. **(A)** Country scientific production; **(B)** The top 10 country with the most publications; **(C)** A visualization to describe the collaborations between countries/regions.

A total of 68 countries/regions contributed to the publications (**Figures 2A, B**). The United States ranked first in the number of publications (n = 485, 17.6%), followed by China (n = 436, 15.8%), the United Kingdom (n = 222, 8%), Japan (n = 201, 7.3%), and Germany (n = 186, 6.7%). The top ten countries accounted for 78.6% of all the publications.

Single-country publications (SCP) refer to papers with co-authors from the same country/region. Multiple country publications (MCP) refer to papers with co-authors from different countries/regions. The United States had the most SCP, MCP, and total citations. The United Kingdom was the leader in terms of the ratio of MCP (MCP-Ratio) to the country’s total publications. Relatively speaking, Chinese articles had the lowest average number of citations. The MCP-Ratio in Japan was the lowest, whereas its number of publications ranked fourth.

### Country/region collaboration

Collaborations between countries/regions were visualized (**Figure 2C**). The most frequent collaboration was from the Netherlands to the United Kingdom (n = 56), followed by from the United Kingdom to Italy (n = 48). Of the top five collaborators, the United States cooperated the most with other countries.

### Publication quantity and journal impact

The retrieved articles were published in 557 journals (**Table 1**). Journals with more than 80 published articles included the *International Journal of Radiation Oncology - Biology - Physics* (n = 88) and *Head and Neck* (n = 86). According to the latest journal citation report of 2023, the *Journal of Clinical Oncology* had the highest impact factor (IF = 42.1; JCR-c: Q1). Five of the top ten journals were from the United States. The remaining two were from the United Kingdom, another two from Switzerland, and one from the Netherlands. The top ten journals accounted for 22.8% of the total, and none of them were from Asia.

**Table 1:** The top 10 journals in publications.

Rank	Sources	Articles	Country	JCR-c	IF
1	International Journal of Radiation Oncology Biology Physics	88	UNITED STATES	Q1	6.4
2	Head and Neck-Journal for the Science and Specialties of the Head and Neck	86	UNITED STATES	Q1	2.3
3	BMC Cancer	78	UNITED KINGDOM	Q2	3.4
4	Cancers	70	SWITZERLAND	Q2	4.5
5	Supportive Care in Cancer	65	UNITED STATES	Q2	2.8
6	Oral Oncology	62	UNITED KINGDOM	Q1	4.0
7	Radiotherapy and Oncology	55	NETHERLANDS	Q2	4.9
8	Frontiers in Oncology	47	SWITZERLAND	Q2	3.5
9	Journal of Clinical Oncology	42	UNITED STATES	Q1	42.1
10	Annals of Surgical Oncology	38	UNITED STATES	Q2	3.4

The impact factors referred to the latest journal citation reports in 2023.

**Figure 3:**
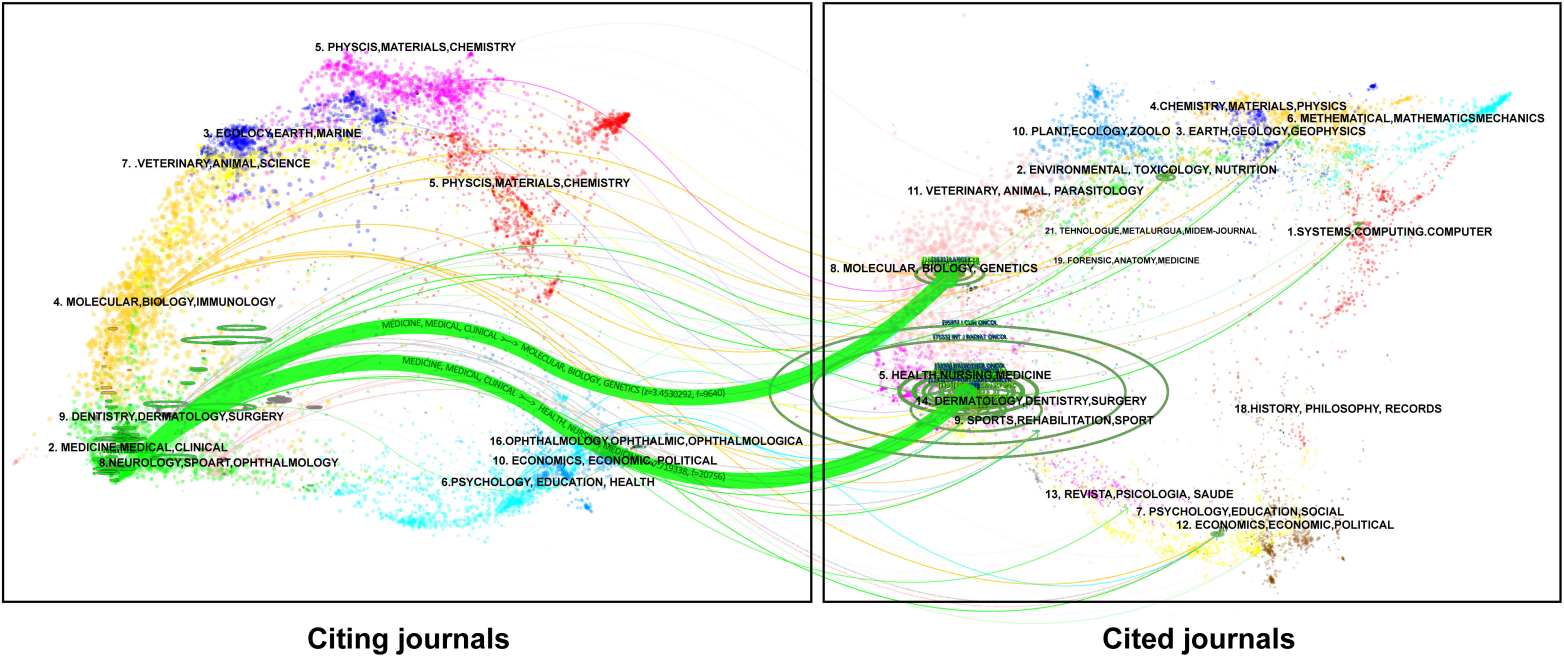
Dual map overlay of journals.

The double map delineated the citation relationships among journals, including citing journals (left) and cited journals (right) (**Figure 3**). The labels represented the topics covered by the journals. Colored curves indicated citation paths, starting from the left and pointing to the right. The main path was from medicine/medical/clinical to health/nursing/medicine papers, followed by that from medicine/medical/clinical to molecular/biology/genetics papers. The two paths exhibited a converging trend.

### Author influence and collaboration

Among 16,147 authors in the selected papers, the most productive were van Berge Henegouwen (n = 27), Wijnhoven (n = 25), and Everett Vokes (n = 21). The H-index was used to assess the level of academic output of the researchers. Van Berge Henegouwen and Vokes topped the H-index, indicating that their influence and contribution to academia were significant. The research topics of the top ten authors were listed in **Supplementary Table S3**.

**Figure 4:**
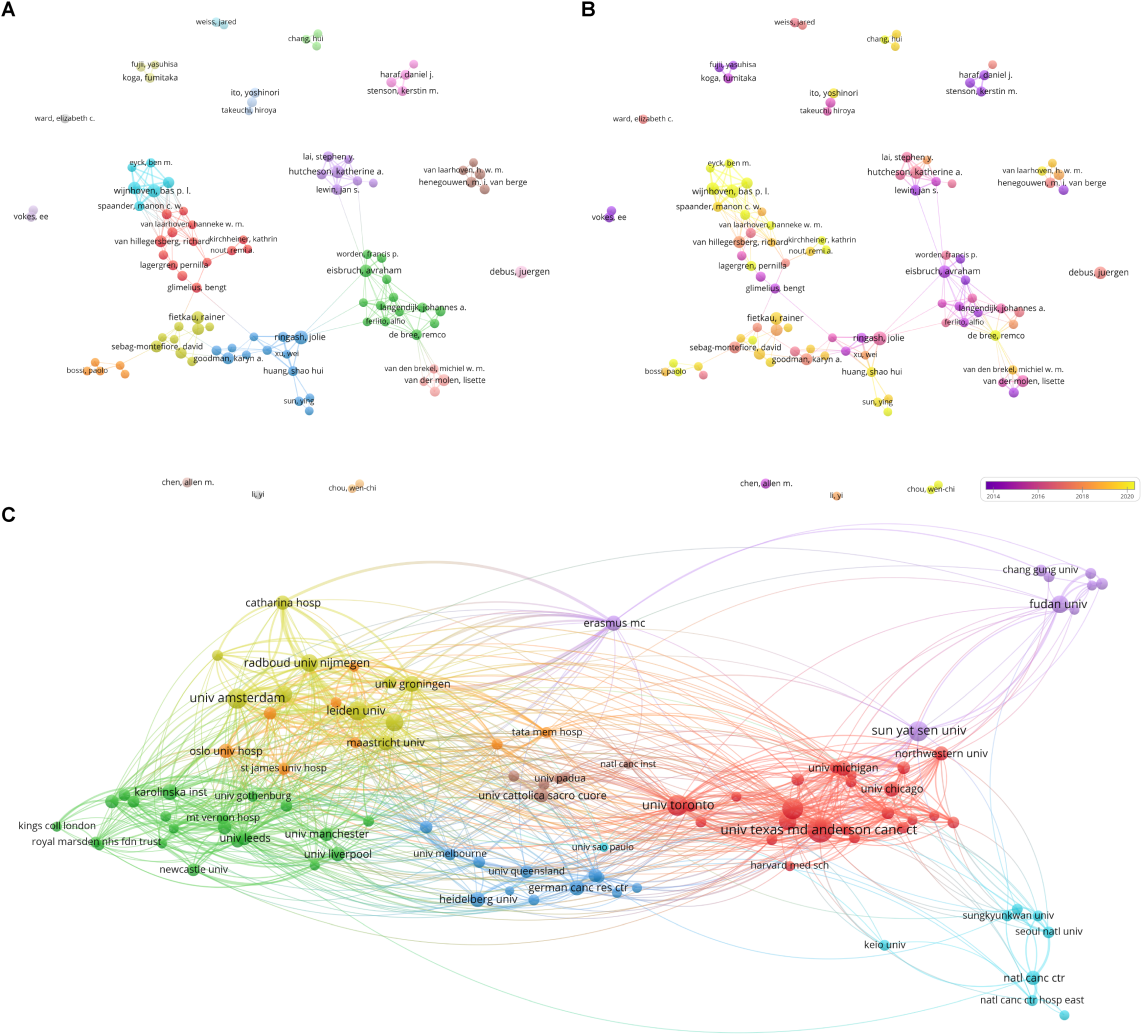
Analysis of co-authorship. **(A)** Network visualization of co-authorship of authors; **(B)** Overlay visualization of co-authorship of authors; **(C)** Network visualization of co-authorship of institutions.

Collaborative relationships among the authors were analyzed through VOSviewer, as shown (**Figures 4A, B**), which presented the relationships between authors, consisting of 100 authors with at least seven publications and 32 citations. The results generated 20 clusters, including six large (n > 5) and 14 small (n ≤ 5) ones. As demonstrated in the time-overlapping map for co-authorship (**Figure 4B**), the cluster centered on Wijnhoven focused on new research in the field.

### Institutional output and collaboration

The analysis identified 2551 institutions. The top ten productive institutions were listed in **Table 2**. The most productive institutions were the University of Toronto in Canada (n = 229), University of Texas MD Anderson Cancer Center in the United States (n = 199), and Unicancer in France (n = 196). In addition, four institutions in the Netherlands and one in China were among the top ten.

**Table 2:** The top 10 institutions with the most publications.

Rank	Institution	Articles	Country
1	University of Toronto	229	CANADA
2	UTMD Anderson Cancer Center	199	UNITED STATES
3	Unicancer	196	FRANCE
4	University of TEXAS System	177	UNITED STATES
5	University of Amsterdam	168	NETHERLANDS
6	Erasmus MC	159	NETHERLANDS
7	SUN YAT SEN University	139	CHINA
8	University Health Network Toronto	137	CANADA
9	Erasmus University Rotterdam	126	NETHERLANDS
10	Utrecht University Medical Center	122	NETHERLANDS

Moreover, institutions with more than 13 publications and 221 citations were selected for visualization analysis to form network visualization (**Figure 4C**), the overlay visualization (**Supplementary Figure S2A**) and the density visualization (**Supplementary Figure S2B**). Upon figures aforementioned, the cooperation among the University of Michigan, University of Chicago, and University of Texas MD Anderson Cancer Centers were conducted early. Consequently, achievements in publication were made which were high production and lasting social impact. In recent years, the number of publications issued by Sun Yat-sen University, Fudan University, and other Chinese institutions had gradually increased, and these institutions had become more active in the field.

### Document hot spots

**Figure 5:**
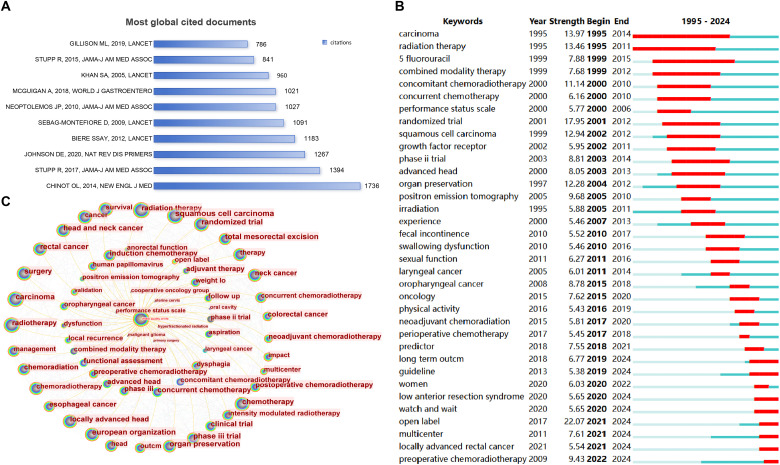
Document analysis and hot spot prediction. **(A)** Most global cited documents; **(B)** Burst detection of keywords; **(C)** Tree-ring map centered on ‘quality of life’.

The cited frequencies of a document can reveal the impact of the research, encompassing local and global citations. Local citations refer to the frequency with which a document is cited in a downloaded dataset. Global citations indicate the number of times a reference is cited by all the literature in the entire WOS database, showing the global impact of a document. As shown in **Supplementary Table S4**, the ten articles with the highest number of local citations were presented. Additionally, **Figure 5A** along with **Supplementary Table S5** displayed the ten articles that have received the highest number of global citations. Most of the most-cited documents were randomized controlled trials published in high-quality journals. Chinot et al. (24) ranked first with 1736 citations globally.

### Citation bursts

The 25 references with the strongest citation bursts are shown (**Supplementary Figure S3**). The article exhibiting the strongest citation burst was Bahadoer (25). Furthermore, two recent articles had a strength of more than 20 (21.64 and 21.28) (26, 27).

### Keyword bursts

The bursts of keywords reflected changes of research hotspots in the field. As illustrated (**Figure 5B**), ‘open label’ (strength = 22.03) ranked first from 2021 to 2024, followed by ‘preoperative chemoradiotherapy’ from 2022 to 2024 (strength = 9.38), ‘multi center’ from 2021 to 2024 (strength = 7.69), ‘women’ from 2020 to 2024 (strength=6.04), ‘watch and wait’ from 2020 to 2024 (strength=5.91), and ‘locally advanced rectal cancer’ from 2020 to 2024 (strength = 5.55).

### Keyword occurrence and co-occurrence

The analysis retrieved 3871 author keywords. The frequency of keyword occurrences unveiled the research priorities and main topics in the research field. The top 50 author keywords were listed (**Supplementary Table S6**), sorted by their occurrence frequency. Scholars focused on ‘QOL’, ‘chemoradiotherapy’, ‘radiotherapy’, ‘head and neck cancer’, and ‘rectal cancer’.

**Figure 6:**
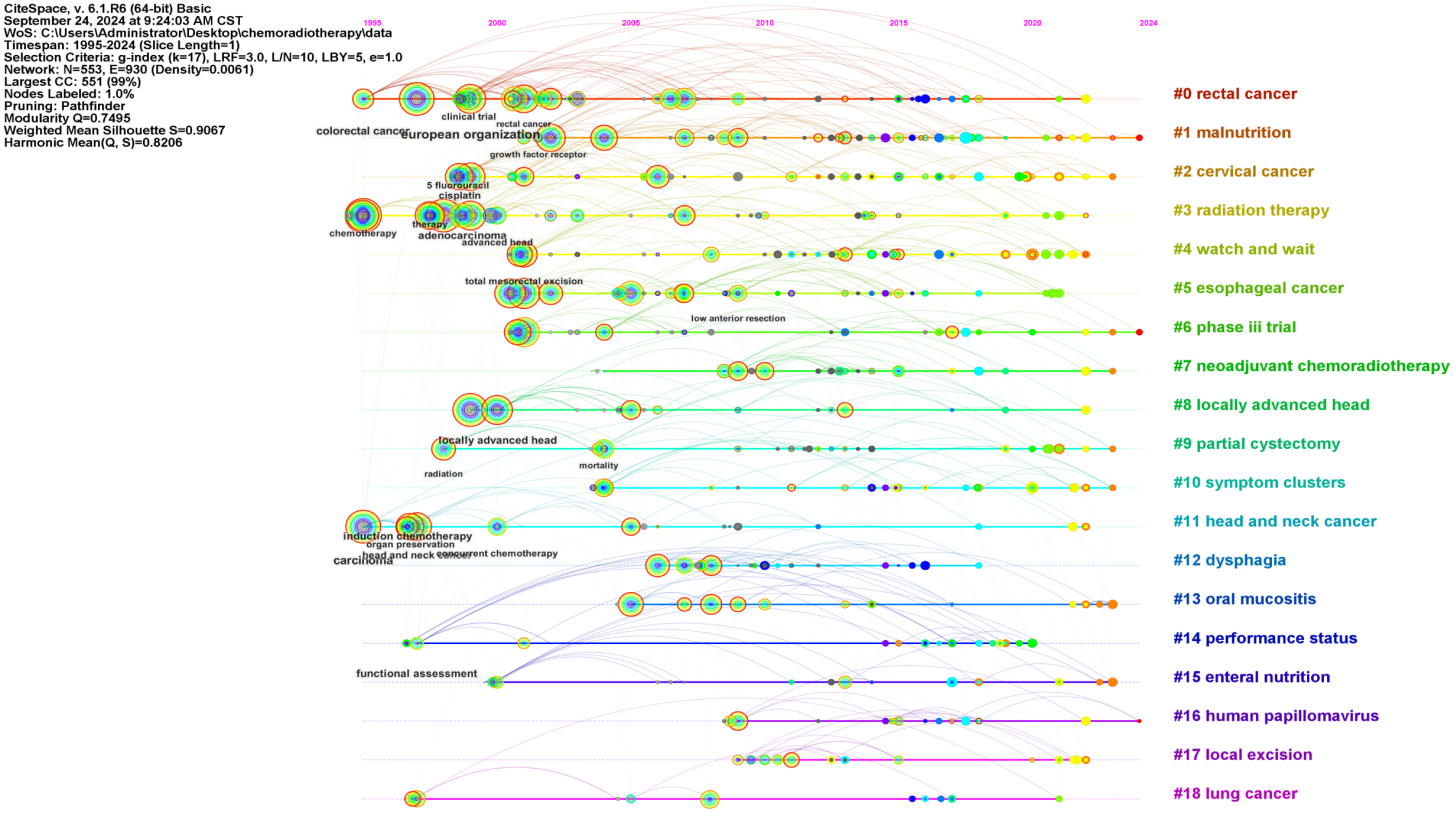
The keyword timeline cluster plot.

A tree-ring map of terms related to QOL was visualized (**Figure 5C**). CiteSpace was used to cluster keywords that had closer relationships through an algorithm and assign a value to each keyword. The keywords with the largest values in the same cluster were selected as representatives of that cluster and used for its label. Additional nodes were included or excluded by adjusting the scale factor k related to the g-index. For better outcomes, cluster analyses were performed when the scale factor k was 17. Consequently, 19 clusters were observed on the keyword timeline cluster map (**Figure 6**). The Q value was 0.7495 and S value was 0.9067, with the Q value indicating the clustering modularity value and S being the average silhouette value of the clusters. Q values > 0.3 indicate that the clustering structure is significant, and S values > 0.7 indicate that the clusters are valid.

**Figure 7:**
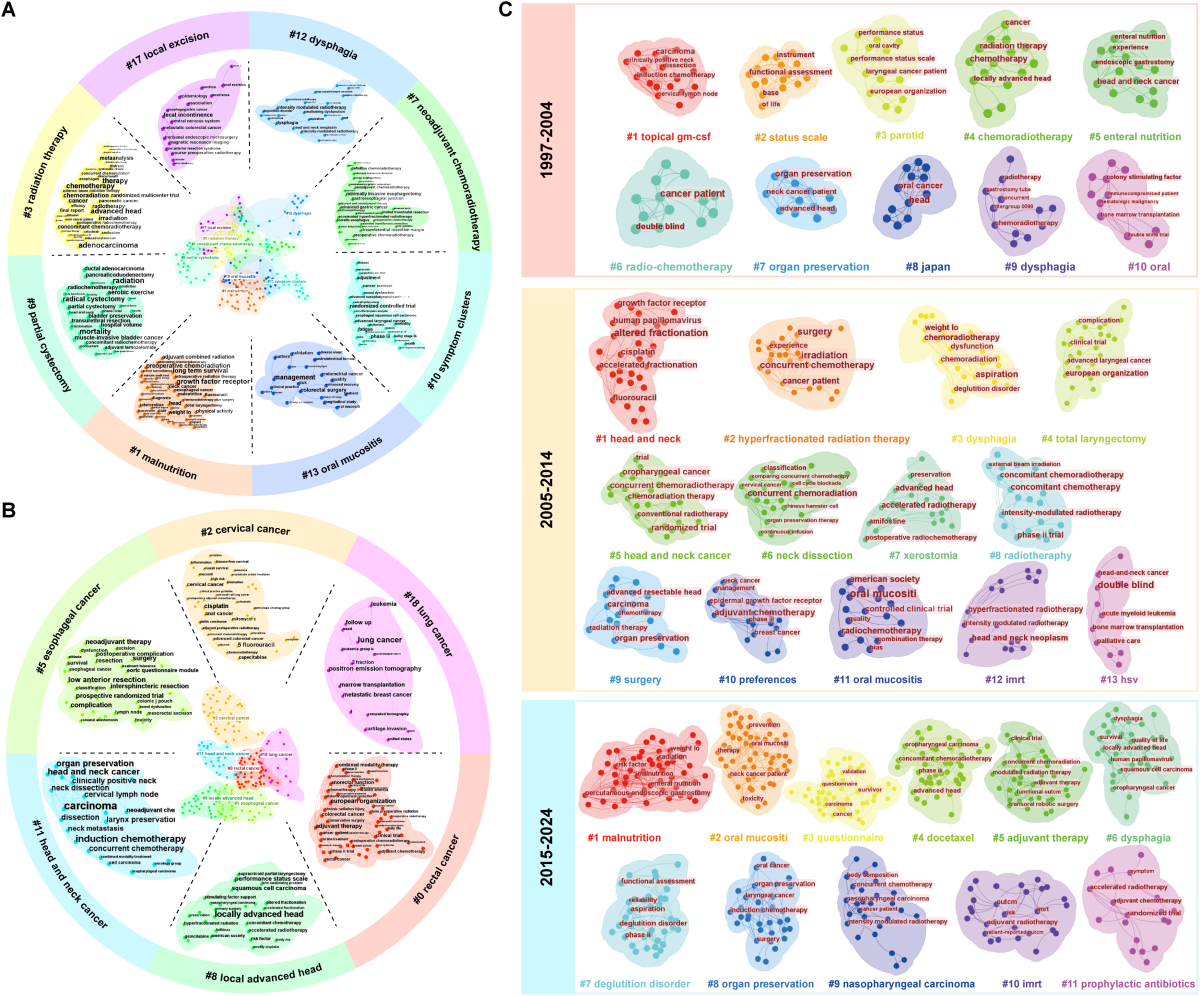
The types of clusters. **(A)** the clusters related to QOL; **(B)** the clusters involving different types of tumors; **(C)** The development of the clusters about head and neck cancer.

Subsequently, in order to carefully observe and comprehend the different features of the 19 clusters, we formed **Figure 7** by classifying these clusters in **Figure 6**. As was shown (**Figures 7A, B**), these clusters were divided into two main categories: one involving different types of tumors including rectal cancer and cervical cancer, and others related to QOL including dysphagia and malnutrition.

The three-field plot was composed of countries, institutions, and keywords, revealing the association and distribution of the three in the field of the QOL of patients with cancer undergoing chemoradiotherapy (**Supplementary Figure S4**). Although differences existed, almost all institutions and countries contributed to the ten themes represented by the keywords. In the national dimension, researchers in the United States covered all ten topics. China focused on ‘dysphagia’ and ‘surgery’, with few studies on ‘survival’. Netherlands also had a high contribution value and focused on ‘oesophageal cancer’. In terms of institutions, the articles from Sun Yat-sen University in China had covered most of the research direction; however, it produced `few publications on ‘oesophageal cancer’.

### Tumor types

**Figure 8:**
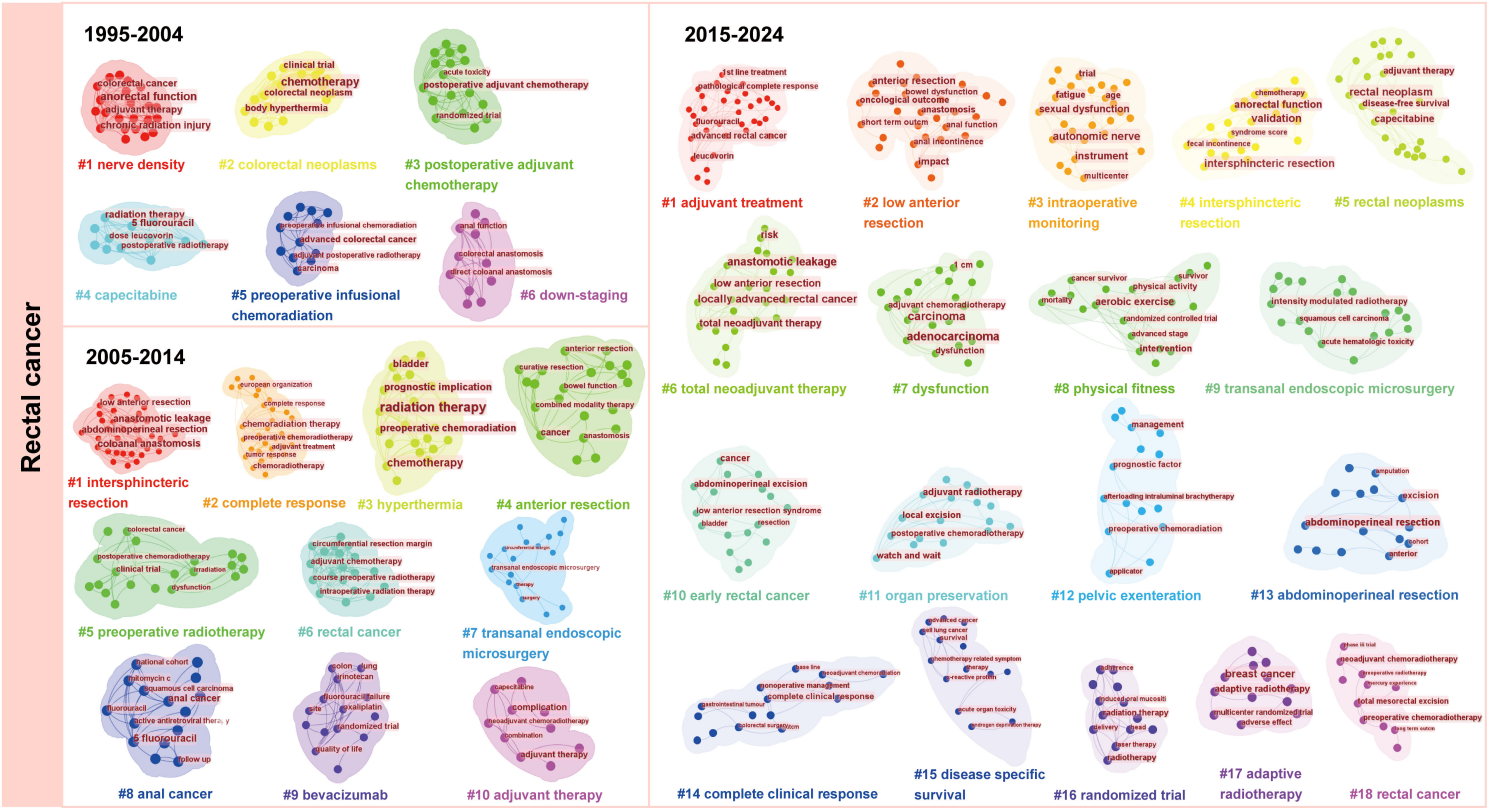
The development of the clusters about rectal cancer.

Keywords regarding head and neck cancer and rectal cancer were clustered due to their highest frequencies (**Supplementary Table S6**). We revealed the change of research focus in this field through the evolution of clusters per decade. The results of cluster analysis of head and neck tumors and rectal cancer were presented (**Figure 7C**, **8**), respectively. From 1997 to 2024, research on head and neck cancer have evolved from focusing on treatment (such as clusters: chemoradiotherapy) and side effects (such as parotid), to an in-depth exploration of treatment techniques (such as hyperfractionated radiation therapy and total laryngectomy), and finally to emphasizing the overall condition (including malnutrition and risk factor) and quality of life of patients (referring to survivor questionnaire). However, the clusters of dysphagia and oral mucositis was observed in all three stages of the study.

Similar to the research on head and neck cancer, researches on rectal cancer focus on the effect of postoperative adjuvant therapy and the impact of preoperative chemoradiotherapy on patient stage, and explore how to improve the treatment effect and survival rate at the first decade. Then, from 2005 to 2014, the researchers are devoted to improving surgical techniques and applying neoadjuvant chemotherapy. Most research from 2015 to 2024 is about integrated treatment and individualized strategies.

Furthermore, a cluster analysis of keywords regarding the remaining tumor types, including cervical cancer, anal cancer, glioblastoma, and bladder cancer, was conducted to explore their development and clusters that affected the outcomes were manually removed. (**Supplementary Figures S5**, **S6**). For anal cancer, the cluster, anorectal function, has been involved throughout the three research stages. After cluster analysis, we observed special cluster phenomena: cervical cancer patients showed an “anemia” cluster, bladder cancer patients had a “gastrointestinal toxicity” cluster, and glioblastoma patients had “melancholia” and “hydrocephalus” clusters. Research on bladder cancer, glioblastoma, and anal cancer was conducted relatively late and developed slowly compared with the other types of cancer that we chose.

### Symptom prevalence and clusters in cervical cancer

Among 117 cervical cancer patients, decreased appetite (79.5%), diarrhea (65.81%), and gustatory alteration (59.0%) were the most prevalent symptoms and also the most severe. Louvain clustering identified three distinct symptom clusters:

1. Cluster 1: xerostomia, numbness/tingling, burping, diarrhea, abdominal pain, hypomnesia, myalgia, vaginal bleeding.
2. Cluster 2: Dizziness, bloating, constipation, alopecia, frequent urination, painful urination, hot flashes and night sweats.
3. Cluster 3: Insomnia, fatigue, decreased libido, altered taste, decreased appetite, nausea and vomiting, dry skin, hyperpigmentation.

The general demographic information of the patients, along with the classification of symptom clusters, is provided in **Supplementary Material** (**Supplementary Tables S7**, **S8**).

## Discussion

With the widespread use of concurrent chemoradiotherapy, an increasing number of cancer patients have survived, attracting the extensive attention of many scholars and researchers. And they began to shift the focus of their research to the QOL of these survivors, trying to understand the various challenges and needs they faced in their recovery. Thus, a bibliometric analysis of 2762 articles on the QOL of patients with cancer undergoing chemoradiotherapy between 1995 and 2024 was conducted. The growth in the annual number of publications showed an exponential trend. The number of accumulated articles was exceeded 1000 in 2016. The growth of articles was slow from 2019 to 2020, perhaps because some studies have stalled during the COVID-19 pandemic. After that, the number of publications reached its highest in 2022 with 266 articles, suggesting that more people may focus on QOL of patients with cancer undergoing chemoradiotherapy in the recent years. The reason may be modern medicine emphasizes that patients should not only survive, but also live with high QOL.

Eighty percent of the total publications came from the top 10 countries. The United States and China, which benefited from their richness in medical resources and emphasis on the medical industry, made the greatest contributions with over 400 publications each. In addition, the average article and total citations of publications from the United Kingdom and Netherlands indicated that their medical technology and research levels were at the forefront of the world. Moreover, collaboration between them was most frequent, suggesting that regular collaboration could facilitate the creation of high-quality articles. Although Asian countries had advantage in publication numbers, the low number of citations of their articles indicated their articles did not seem to be recognized by global researchers.

The Netherlands was among the top ten countries in terms of publications and had a high average number of article citations; unsurprisingly, seven of the top ten productive authors were from the Netherlands. Almost all of the top ten authors formed a close network of partnerships centered on themselves. This not only strengthened the links and cooperation between authors but also contributed to the continuous deepening and advancement of research in the field.

The models of cooperation between institutions were similar, including government–university, university–university, and government–business cooperation. Sun Yat-sen University in China ranked seventh in terms of publications, with a large gap from the top position. Furthermore, several institutions from China recently formed a network of cooperation with strong potential. Moreover, the largest cooperative cluster comprised University of Michigan, University of Chicago, and University of Texas MD Anderson Cancer Center. The three institutions in this field started early, were closely related, and achieved remarkable results. Analyses from the perspective of countries, institutions, and authors indicate that there is a need to promote international exchange and cooperation.

The journal with the largest number of documents is *International Journal of Radiation Oncology - Biology - Physics*, with 88 accepted documents. Half of the top journals come from the United States. Usually, scholars use the IF as an evaluation of the quality and influence of a journal. The IF of the top 10 journals were above 2.0, with *Journal of Clinical Oncology* having the highest one. The citation status of journals is consistent with the evolution of the discipline: as medicine advances, it moves from organs to cells to molecules and genes, with micro-level studies in turn providing evidence for treatment and contributing to human health.

This study aimed to derive hotspots (22) through keyword burst detection and identify the research topics of the most cited articles. As the red parts represent keywords with strong citation bursts, it’s easy to get the hotspots from the keywords burst detection. Between 1995 and 2004, research focused on ‘radiation therapy’, ‘irradiation’, ‘5- fluorouracil’, ‘combined modality therapy’, and ‘concurrent chemoradiotherapy’. Researchers during this period may have realized the therapeutic efficacy of combining radiotherapy and chemotherapy (28). Consequently, they explored specific treatment options for concurrent chemoradiotherapy for patients with cancer, including medication, medication dosage, and methods of combining therapies (9). Between 2005 and 2014, concurrent chemoradiotherapy gradually became the mainstream cancer treatment. Researchers began to seek the optimal treatment protocols for patients with various cancer types and stages (29) and established clinical practice guidelines for concurrent chemoradiotherapy. Thus, ‘advanced laryngeal cancer’, ‘laryngeal cancer’, ‘oropharyngeal cancer’, and ‘oncology’ hotspots appeared during this phase. Subsequently, several topics have gained attention, including ‘long term outcome’, ‘women’, ‘watch and wait’, ‘open label’, ‘multi center’, ‘predictor’ and ‘total neoadjuvant therapy’. It suggests that as health care providers and patients began focusing on long-term outcomes, QOL is gradually being valued (30) during the treatment process.

The transition of research hotspots from treatment effect to patient experience and long-term prognosis in the treatment process is attributable to multiple factors. First of all, the global increase in cancer burden and the expanding number of survivors have further necessitated a focus on survivorship issues (31), making patient experience and long-term prognosis crucial research areas. Then, the rise of emerging treatment concepts has played an important role. For example, personalized treatment strategies (32), which take patients’ experience and treatment outcomes into consideration, as well as holistic care focusing on the physical (31), psychological and social well-being of patients. Meanwhile, as patients are gradually and deeply involved in medical decision making, it is fully reasonable and inevitable to bring quality of life into the scope of treatment. Finally, technological innovations such as intensity-modulated radiotherapy and minimally invasive surgical techniques have emerged continuously. The application of these innovative technologies in clinical practice has led to a change in research directions. Researchers have begun to explore in depth their impacts on patient experience and long-term treatment outcomes, further facilitating the shift of research hotspots.

Similar to keyword burst detection, almost all articles and references referred to new therapies, such as intensity-modulated radiotherapy, minimally invasive surgeries and neoadjuvant treatments. In 2010, Feng et al. (33) conducted a prospective study on the use of intensity-modulated radiotherapy to preserve important swallowing structures and reduce post-therapy dysphagia. They anticipated that if they included only lateral retropharyngeal nodes in the tumor targets and excluded medial ones that were close to the tumor targets but rarely metastasised, they could protect pharyngeal constrictors without increasing the risk of local tumor recurrence. Their study demonstrated that intensity-modulated radiotherapy was effective for organ preservation; however, some patients experienced new problems that required follow-up intervention.

Comprehensive cancer treatments include radiotherapy, chemotherapy, and surgical resection. Therefore, many researchers have also examined surgical methods. In 2012, the *Lancet* published an article that compared minimally invasive and open oesophagectomy (34). Minimally invasive oesophagectomy was superior to open surgery in reducing the rate of postoperative lung infection, shortening hospitalization, and improving short-term QOL. This study argued that the long-term outcomes of minimally invasive surgeries should be considered.

The role of neoadjuvant treatments is becoming increasingly prominent. With these treatments, patients undergo chemoradiotherapy before surgery to lower the cancer stage and increase the likelihood of surgical resection (35). This method was initially not favored by researchers, as it did not improve survival and could increase postoperative morbidity and mortality (36). However, with continued research and development of new medications, neoadjuvant therapy has made remarkable progress in multiple oncological fields (37). Neoadjuvant therapy emphasizes individualized treatment and plans according to the patient’s condition and tumor characteristics (38), which improves the treatment effect and ensures that patients are able to maintain a high QOL.

Pan-cancer Research is a research method designed to understand the common features between different types of cancer. The subsequent cluster analysis of pan-cancer we conducted demonstrated that research has increasingly focused on patient fitness, dysfunction and quality of life, reflecting the contemporary medical tradition of emphasizing the importance of personalized treatment and the patient experience, which demonstrates a patient-centered philosophy. Furthermore, there are certain commonalities that can be identified. The primary site of all tumor types suitable for chemoradiotherapy was the hollow viscus. Squamous cell carcinoma had high specificity for concurrent chemoradiotherapy.

Patients with these tumors face a common challenge that affects their QOL, namely organ preservation (39). Radical surgery is the oldest treatment for cancer (40). However, patients face a complex and critical issue: each organ has a unique function, and the loss of any one can have a profound effect on an individual’s QOL (41). Patients have to deal with not only physical deficiencies but also the resulting psychological distress. A self-image disorder is a common psychological response to organ loss (42). Thus, physicians should focus on organ preservation during cancer treatment to maintain positive patient QOL (34).

Moreover, special factors affect the QOL of patients with different tumor types undergoing concurrent chemoradiotherapy. So for specific tumor types, such as head and neck cancer, rectal cancer, anal cancer, cervical cancer, bladder cancer and glioblastoma, we have carried out in-depth investigations. The presence of dysphagia and oral mucositis throughout three research stages of head and neck cancer indicates that deglutition disorder and oral mucositis has consistently been a significant factor impacting these patients’ QOL (43). This treatment complication not only affects feeding and nutrition absorption (44) but may also aggravate the psychological burden. The development of neoadjuvant therapy in recent years has promoted the widespread application of the watch and wait strategy for rectal cancer (27). This strategy is implemented for rectal cancer patients who have achieved a complete clinical response after neoadjuvant therapy, aiming to reduce unnecessary treatments, relieve the physical burden on patients, and ultimately improve the quality of life of patients (45). In anal cancer, anorectal function continues to be a focus of attention, it is clear that preserving anal sphincter function is a key aspect in improving QOL (46). During the process of chemoradiotherapy for bladder cancer, in addition to causing symptoms of urinary system dysfunction (47) such as frequent urination, urgent urination, painful urination and hematuria, it is often accompanied by gastrointestinal toxicity reactions. Similarly, in the treatment of glioblastoma patients, special attention should also be paid to melancholia symptoms and the patients’ conditions. The Karnofsky (48) scale is commonly used to continuously monitor their functions and conditions, so as to prevent hydrocephalus and avoid the decline of QOL (49). Notably, the symptom experienced by cervical cancer patients undergoing concurrent chemoradiotherapy have become a focal point in clinical research. Current evidence demonstrates that symptom and symptom clusters may impair the quality of life (QOL) of patients. Zhang et al. (10) used the M. D. Anderson Symptom Inventory (MDASI) and found that the five most commonly occurring symptoms were fatigue (80.8%), disturbed sleep (79.7%), pain (76.9%), sadness (75.5%), and nausea (73.4%). They also identified four clusters of symptoms: adverse effects of therapy, sickness, gastrointestinal and psychological status contributed 22.4% to the total. And the symptom of anemia seems to be particular, requiring attention throughout the medical attention and intervention process. In contrast, our study, based on the CTCAE 5.0, revealed that decreased appetite, diarrhea, and altered taste were among the most frequent and severe symptoms, emphasizing the critical role of nutritional management in early-phase CCRT interventions. This difference not only stems from the inherent characteristics of the evaluation tools, but more fundamentally reflects the unique clinical needs for individualized symptom management strategies after CCRT. In the clinical context of cervical cancer concurrent chemoradiotherapy (CCRT), where treatment toxicity is the predominant factor, CTCAE 5.0 demonstrates greater practical value in symptom monitoring. Additionally, in terms of treatment, neoadjuvant chemoradiotherapy serves as a valuable complement to concurrent chemoradiotherapy. Concurrent chemoradiotherapy combined with immunotherapy also provides novel treatment options, such as the use of NK cells in the treatment of gliomas. Therefore, identifying these factors not only facilitates more precise medical care provided by healthcare professionals but also stimulates the development of novel treatment strategies.

## Limitations

This study had several limitations. Firstly, as this was a bibliometric analysis, data processing may be affected by the software used. The reliability of the analysis results can be verified by comparing the data analysis results of multiple software. Secondly, we searched only the WOSCC database and included articles written in English. It may make us fail to understand the research from a multicultural perspective. We will follow up with an ongoing search for articles in this area for best results. Additionally, despite our comprehensive search strategy, which encompassed both general and specific cancers, it is possible that some relevant literature may have been overlooked. Finally, the relatively limited sample size may have restricted the generalizability of the research findings to broader populations. Future studies should involve multicenter, large-scale cohort studies to further validate the current findings.

## Conclusion

The impact of concurrent chemoradiotherapy on the QOL of patients is garnering increasing attention. Studies have shown that more importance is attached to the patient experience and long-term health consequences in cancer treatment. Through conducting analyses of pan-cancer and different cancer types, the research has identified multiple factors that affect the quality of life of patients undergoing radiotherapy and chemotherapy, including treatment methods, treatment-induced symptoms (such as nutritional issues), psychological factors and so on. Overall, this study has provided key insights into understanding the impact of radiotherapy and chemotherapy on the quality of life and has provided useful guidance for future research and clinical practice in this field.

## Data Availability

The datasets presented in this study can be found in online repositories. The names of the repository/repositories and accession number(s) can be found in the article/**Supplementary Material**.

## Supplementary material

Supplementary Figure 1 Analysis of annual scientific production. **(A)** the main information of the included articles; **(B)** the annual and cumulative publications.

Supplementary Figure 2 Co-authorship analysis of citations. **(A)** Overlay visualization of co-authorship of institutions; **(B)** Density of co-authorship of institutions. It displayed a visualization of institution density, with labels for institutions and color indicating document density. Higher article counts meant greater weight and closer to yellow color.

Supplementary Figure 3 Burst detection of citations. Blue indicates time and dark blue indicates the time period that a reference appears. Besides, Red part represents the time of citation burst.

Supplementary Figure 4 Three-field plot of the authors’ keywords analysis (Left field: countries; Middle field: keywords; Right field: institutions).

Supplementary Figure 5 Clusters analysis of anal cancer and cervical cancer. **(A)** The development of the clusters about anal cancer; **(B)** The development of the clusters about cervical cancer.

Supplementary Figure 6 Clusters analysis of bladder cancer and glioblastoma. **(A)** The development of the clusters about bladder cancer; **(B)** The development of the clusters about glioblastoma.

## References

[1] Bray F Laversanne M Weiderpass E Soerjomataram I. The ever-increasing importance of cancer as a leading cause of premature death worldwide. Cancer. (2021) 127:3029–30. doi: 10.1002/cncr.33587, PMID: 34086348

[2] Bray F Laversanne M Sung H Ferlay J Siegel RL Soerjomataram I. Global cancer statistics 2022: GLOBOCAN estimates of incidence and mortality worldwide for 36 cancers in 185 countries. CA A Cancer J Clin. (2024) 74:229–63. doi: 10.3322/caac.21834, PMID: 38572751

[3] Chen S Cao Z Prettner K Kuhn M Yang J Jiao L. Estimates and projections of the global economic cost of 29 cancers in 204 countries and territories from 2020 to 2050. JAMA Oncol. (2023) 9:465. doi: 10.1001/jamaoncol.2022.7826, PMID: 36821107 PMC9951101

[4] Tang L-L Guo R Zhang N Deng B Chen L Cheng Z-B. Effect of radiotherapy alone vs radiotherapy with concurrent chemoradiotherapy on survival without disease relapse in patients with low-risk nasopharyngeal carcinoma: A randomized clinical trial. JAMA. (2022) 328:728. doi: 10.1001/jama.2022.13997, PMID: 35997729 PMC9399866

[5] Abu-Rustum NR Yashar CM Arend R Barber E Bradley K Brooks R. NCCN guidelines^®^ Insights: cervical cancer, version 1.2024: featured updates to the NCCN guidelines. J Natl Compr Cancer Network. (2023) 21:1224–33. doi: 10.6004/jnccn.2023.0062, PMID: 38081139

[6] Caudell JJ Gillison ML Maghami E Spencer S Pfister DG Adkins D. NCCN guidelines^®^ Insights: head and neck cancers, version 1.2022: featured updates to the NCCN guidelines. J Natl Compr Cancer Network. (2022) 20:224–34. doi: 10.6004/jnccn.2022.0016, PMID: 35276673

[7] Benson AB Venook AP Bekaii-Saab T Chan E Chen Y-J Cooper HS. Rectal cancer, version 2.2015. J Natl Compr Canc Netw. (2015) 13:719–28. doi: 10.6004/jnccn.2015.0087, PMID: 26085388

[8] Firkins J Hansen L Driessnack M Dieckmann N. Quality of life in “chronic” cancer survivors: a meta-analysis. J Cancer Surviv. (2020) 14:504–17. doi: 10.1007/s11764-020-00869-9, PMID: 32162194

[9] Gillison ML Trotti AM Harris J Eisbruch A Harari PM Adelstein DJ. Radiotherapy plus cetuximab or cisplatin in human papillomavirus-positive oropharyngeal cancer (NRG Oncology RTOG 1016): a randomised, multicentre, non-inferiority trial. Lancet. (2019) 393:40–50. doi: 10.1016/S0140-6736(18)32779-X, PMID: 30449625 PMC6541928

[10] Zhang L Wang J Chen T Tian M Zhou Q Ren J. Symptom clusters and quality of life in cervical cancer patients receiving concurrent chemoradiotherapy: the mediating role of illness perceptions. Front Psychiatry. (2022) 12:807974. doi: 10.3389/fpsyt.2021.807974, PMID: 35173639 PMC8841507

[11] Staquet M Berzon R Osoba D Machin D. Guidelines for reporting results of quality of life assessments in clinical trials. Qual Life Res. (1996) 5:496–502. doi: 10.1007/BF00540022, PMID: 8973129

[12] Murrell R. Quality of life and neurological illness: a review of the literature. Neuropsychol Rev. (1999) 9(4):209–29. doi: 10.1023/a:1021686606648, PMID: 10667448

[13] Hamming JF De Vries J. Measuring quality of life. Br J Surg. (2007) 94:923–4. doi: 10.1002/bjs.5948, PMID: 17636515

[14] Karimi M Brazier J. Health, health-related quality of life, and quality of life: what is the difference? PharmacoEconomics. (2016) 34:645–9. doi: 10.1007/s40273-016-0389-9, PMID: 26892973

[15] Chiu L Chiu N Chow E Cella D Beaumont JL Lam H. Comparison of three shortened questionnaires for assessment of quality of life in advanced cancer. J Palliative Med. (2014) 17:918–23. doi: 10.1089/jpm.2014.0012, PMID: 24922521

[16] Wilson TR Birks Y Alexander DJ. Pitfalls in the interpretation of standardised quality of life instruments for individual patients? A qualitative study in colorectal cancer. Qual Life Res. (2013) 22:1879–88. doi: 10.1007/s11136-012-0303-7, PMID: 23135923

[17] Donthu N Kumar S Mukherjee D Pandey N Lim WM. How to conduct a bibliometric analysis: An overview and guidelines. J Business Res. (2021) 133:285–96. doi: 10.1016/j.jbusres.2021.04.070

[18] De Winter JCF Zadpoor AA Dodou D. The expansion of Google Scholar versus Web of Science: a longitudinal study. Scientometrics. (2014) 98:1547–65. doi: 10.1007/s11192-013-1089-2

[19] Yeung AWK. Comparison between scopus, web of science, pubmed and publishers for mislabelled review papers. Curr Sci. (2019) 116:1909. doi: 10.18520/cs/v116/i11/1909-1914

[20] Wu F Gao J Kang J Wang X Niu Q Liu J. Knowledge mapping of exosomes in autoimmune diseases: A bibliometric analysis (2002–2021). Front Immunol. (2022) 13:939433. doi: 10.3389/fimmu.2022.939433, PMID: 35935932 PMC9353180

[21] Arruda H Silva ER Lessa M Proença D Bartholo R. VOSviewer and bibliometrix. jmla. (2022) 110:392–5. doi: 10.5195/jmla.2022.1434, PMID: 36589296 PMC9782747

[22] Chen C Hu Z Liu S Tseng H. Emerging trends in regenerative medicine: a scientometric analysis in. CiteSpace Expert Opin Biol Ther. (2012) 12:593–608. doi: 10.1517/14712598.2012.674507, PMID: 22443895

[23] Van Eck NJ Waltman L. Citation-based clustering of publications using CitNetExplorer and VOSviewer. Scientometrics. (2017) 111:1053–70. doi: 10.1007/s11192-017-2300-7, PMID: 28490825 PMC5400793

[24] Chinot OL Wick W Mason W Henriksson R Saran F Nishikawa R. Bevacizumab plus radiotherapy–temozolomide for newly diagnosed glioblastoma. N Engl J Med. (2014) 370:709–22. doi: 10.1056/NEJMoa1308345, PMID: 24552318

[25] Bahadoer RR Dijkstra EA Van Etten B Marijnen CAM Putter H Kranenbarg EM-K. Short-course radiotherapy followed by chemotherapy before total mesorectal excision (TME) versus preoperative chemoradiotherapy, TME, and optional adjuvant chemotherapy in locally advanced rectal cancer (RAPIDO): a randomised, open-label, phase 3 trial. Lancet Oncol. (2021) 22:29–42. doi: 10.1016/S1470-2045(20)30555-6, PMID: 33301740

[26] Conroy T Bosset J-F Etienne P-L Rio E François É Mesgouez-Nebout N. Neoadjuvant chemotherapy with FOLFIRINOX and preoperative chemoradiotherapy for patients with locally advanced rectal cancer (UNICANCER-PRODIGE 23): a multicentre, randomised, open-label, phase 3 trial. Lancet Oncol. (2021) 22:702–15. doi: 10.1016/S1470-2045(21)00079-6, PMID: 33862000

[27] Van Der Valk MJM Hilling DE Bastiaannet E Meershoek-Klein Kranenbarg E Beets GL Figueiredo NL. Long-term outcomes of clinical complete responders after neoadjuvant treatment for rectal cancer in the International Watch & Wait Database (IWWD): an international multicentre registry study. Lancet. (2018) 391:2537–45. doi: 10.1016/S0140-6736(18)31078-X, PMID: 29976470

[28] Nguyen NP Sallah S Karlsson U Antoine JE. Combined chemotherapy and radiation therapy for head and neck Malignancies: Quality of life issues. Cancer. (2002) 94:1131–41. doi: 10.1002/cncr.10257, PMID: 11920484

[29] Fokas E Schlenska-Lange A Polat B Klautke G Grabenbauer GG Fietkau R. Chemoradiotherapy plus induction or consolidation chemotherapy as total neoadjuvant therapy for patients with locally advanced rectal cancer: long-term results of the CAO/ARO/AIO-12 randomized clinical trial. JAMA Oncol. (2022) 8:e215445. doi: 10.1001/jamaoncol.2021.5445, PMID: 34792531 PMC8603234

[30] Neibart SS Manne SL Jabbour SK. Quality of life after radiotherapy for rectal and anal cancer. Curr Colorectal Cancer Rep. (2020) 16:1–10. doi: 10.1007/s11888-019-00448-w, PMID: 32632345 PMC7336840

[31] Frisch NC Rabinowitsch D. What’s in a definition? *Holistic nursing, integrative health care*, and *integrative nursing* : report of an integrated literature review. J Holist Nurs. (2019) 37:260–72. doi: 10.1177/0898010119860685, PMID: 31257971

[32] Jackson SE Chester JD. Personalised cancer medicine. Intl J Cancer. (2015) 137:262–6. doi: 10.1002/ijc.28940, PMID: 24789362

[33] Feng FY Kim HM Lyden TH Haxer MJ Worden FP Feng M. Intensity-modulated chemoradiotherapy aiming to reduce dysphagia in patients with oropharyngeal cancer: clinical and functional results. JCO. (2010) 28:2732–8. doi: 10.1200/JCO.2009.24.6199, PMID: 20421546 PMC2881852

[34] Biere SS Van Berge Henegouwen MI Maas KW Bonavina L Rosman C Garcia JR. Minimally invasive versus open oesophagectomy for patients with oesophageal cancer: a multicentre, open-label, randomised controlled trial. Lancet. (2012) 379:1887–92. doi: 10.1016/S0140-6736(12)60516-9, PMID: 22552194

[35] Scott AJ Kennedy EB Berlin J Brown G Chalabi M Cho MT. Management of locally advanced rectal cancer: ASCO guideline. JCO. (2024) 42:3355–75. doi: 10.1200/JCO.24.01160, PMID: 39116386

[36] Vogt K Fenlon D Rhodes S Malthaner R The Cochrane Collaboration. Preoperative chemotherapy for resectable thoracic esophageal cancer. In: Cochrane Database of Systematic Reviews. John Wiley & Sons, Ltd, Chichester, UK (2006). p. CD001556. doi: 10.1002/14651858.CD001556.pub2, PMID:

[37] Trip AK Poppema BJ Van Berge Henegouwen MI Siemerink E Beukema JC Verheij M. Preoperative chemoradiotherapy in locally advanced gastric cancer, a phase I/II feasibility and efficacy study. Radiother Oncol. (2014) 112:284–8. doi: 10.1016/j.radonc.2014.05.003, PMID: 24856116

[38] Sauer R Liersch T Merkel S Fietkau R Hohenberger W Hess C. Preoperative versus postoperative chemoradiotherapy for locally advanced rectal cancer: results of the German CAO/ARO/AIO-94 randomized phase III trial after a median follow-up of 11 years. JCO. (2012) 30:1926–33. doi: 10.1200/JCO.2011.40.1836, PMID: 22529255

[39] Baird P JD Minnaar HS Stewart AJ. Assessment of quality of life in rectal cancer with organ-preservation treatment: are we there yet? Clin Oncol. (2023) 35:e110–20. doi: 10.1016/j.clon.2022.11.002, PMID: 36443138

[40] Smith JJ Garcia-Aguilar J. Advances and challenges in treatment of locally advanced rectal cancer. JCO. (2015) 33:1797–808. doi: 10.1200/JCO.2014.60.1054, PMID: 25918296 PMC4559608

[41] Forastiere AA Weber RS Trotti A. Organ preservation for advanced larynx cancer: issues and outcomes. JCO. (2015) 33:3262–8. doi: 10.1200/JCO.2015.61.2978, PMID: 26351339 PMC5320920

[42] Garcia-Aguilar J Patil S Gollub MJ Kim JK Yuval JB Thompson HM. Organ preservation in patients with rectal adenocarcinoma treated with total neoadjuvant therapy. JCO. (2022) 40:2546–56. doi: 10.1200/JCO.22.00032, PMID: 35483010 PMC9362876

[43] Johnson DE Burtness B Leemans CR Lui VWY Bauman JE Grandis JR. Head and neck squamous cell carcinoma. Nat Rev Dis Primers. (2020) 6:92. doi: 10.1038/s41572-020-00224-3, PMID: 33243986 PMC7944998

[44] Rosenthal DI Lewin JS Eisbruch A. Prevention and treatment of dysphagia and aspiration after chemoradiation for head and neck cancer. JCO. (2006) 24:2636–43. doi: 10.1200/JCO.2006.06.0079, PMID: 16763277

[45] Smith JJ Strombom P Chow OS Roxburgh CS Lynn P Eaton A. Assessment of a watch-and-wait strategy for rectal cancer in patients with a complete response after neoadjuvant therapy. JAMA Oncol. (2019) 5:e185896. doi: 10.1001/jamaoncol.2018.5896, PMID: 30629084 PMC6459120

[46] Faaborg PM Haas S Liao D Ploen J Jakobsen A Rahr HB. Long-term anorectal function in rectal cancer patients treated with chemoradiotherapy and endorectal brachytherapy. Colorectal Dis. (2021) 23:2311–9. doi: 10.1111/codi.15692, PMID: 33900676

[47] Lenis AT Lec PM Chamie K Mshs M. Bladder cancer: A review. JAMA. (2020) 324:1980. doi: 10.1001/jama.2020.17598, PMID: 33201207

[48] Bauchet L Zouaoui S Darlix A Menjot De Champfleur N Ferreira E Fabbro M. Assessment and treatment relevance in elderly glioblastoma patients. Neuro-Oncology. (2014) 16:1459–68. doi: 10.1093/neuonc/nou063, PMID: 24792440 PMC4201066

[49] MoNtaNo N. Communicating hydrocephalus following surgery and adjuvant radiochemotherapy for glioblastoma. J Neurosurg. (2011) 115(6):1126–30. doi: 10.3171/2011.8.JNS11738, PMID: 21905801

