# Supplementary figures and images for: Quality of life in patients with pan-cancer undergoing concurrent chemoradiotherapy: a bibliometric analysis (1995-2024)
Source: Front Oncol. 2025 Aug 12;15:1572725. doi: 10.3389/fonc.2025.1572725 (PMC12378759; doi:10.3389/fonc.2025.1572725)

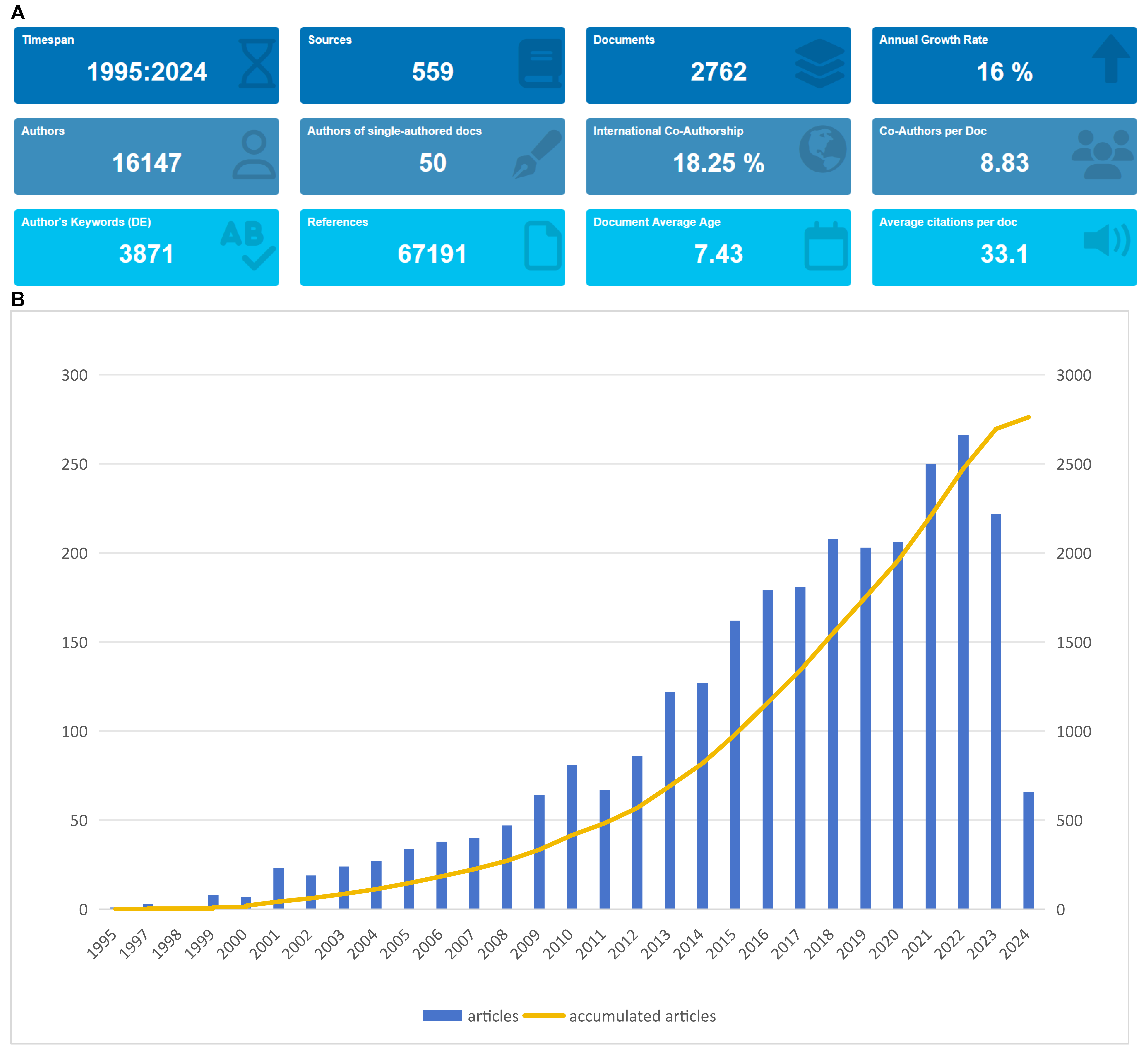

Supplement: Supplementary Figure 1 — Analysis of annual scientific production. (A) the main information of the included articles; (B) the annual and cumulative publications. [file Image1.tif]

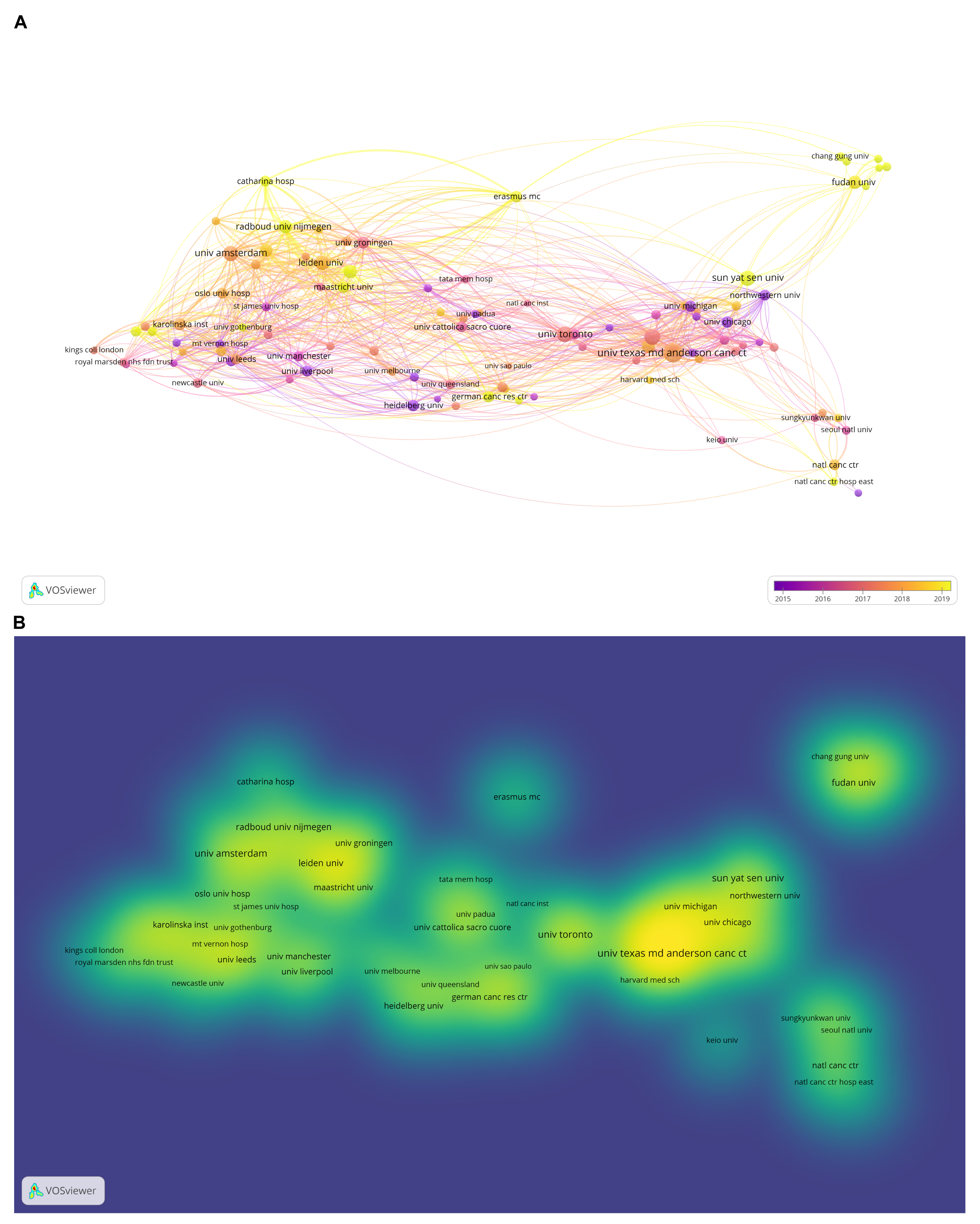

Supplement: Supplementary Figure 2 — Co-authorship analysis of citations. (A) Overlay visualization of co-authorship of institutions; (B) Density of co-authorship of institutions. It displayed a visualization of institution density, with labels for institutions and color indicating document density. Higher article counts meant greater weight and closer to yellow color. [file Image2.tif]

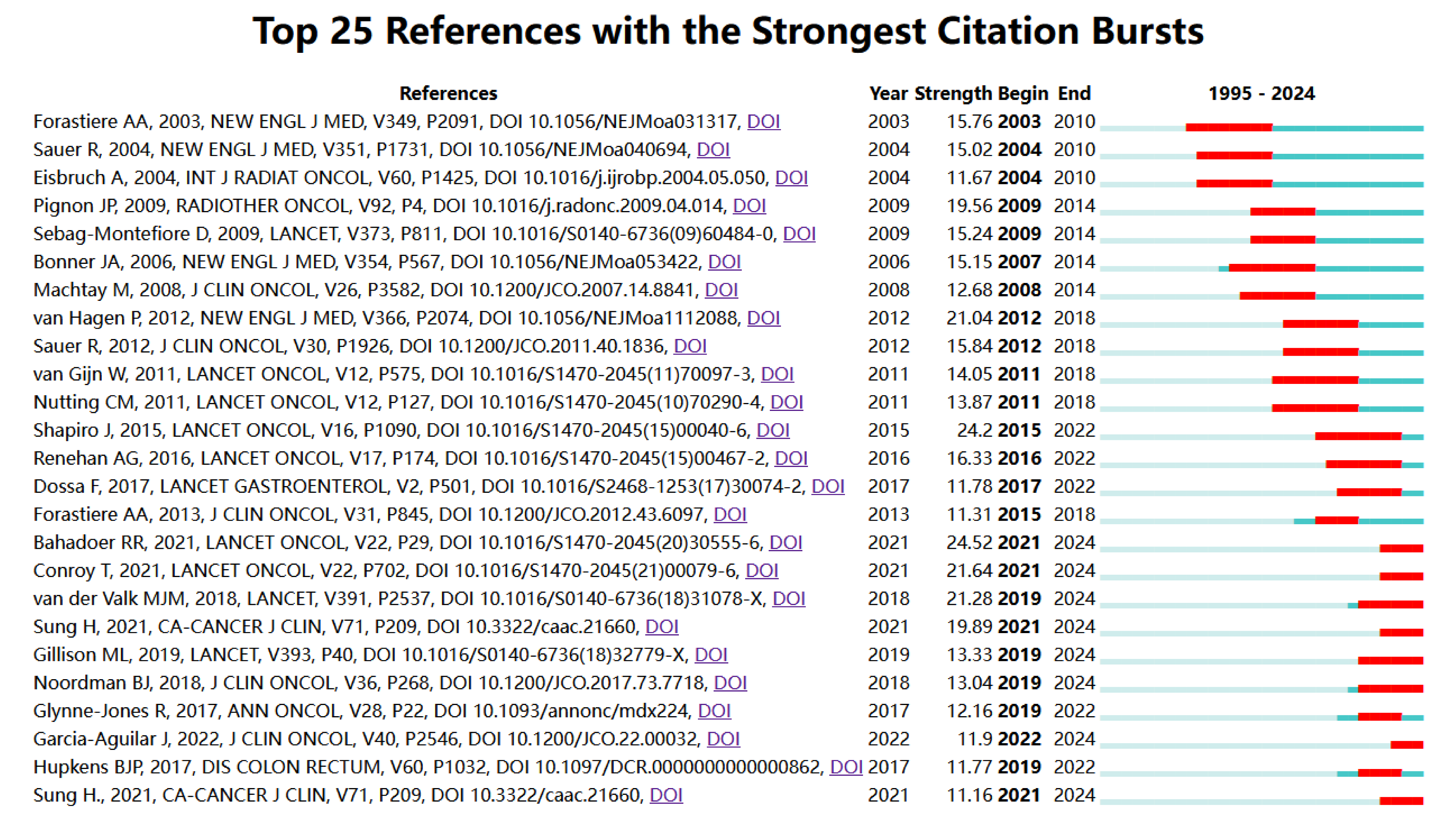

Supplement: Supplementary Figure 3 — Burst detection of citations. Blue indicates time and dark blue indicates the time period that a reference appears. Besides, Red part represents the time of citation burst. [file Image3.tif]

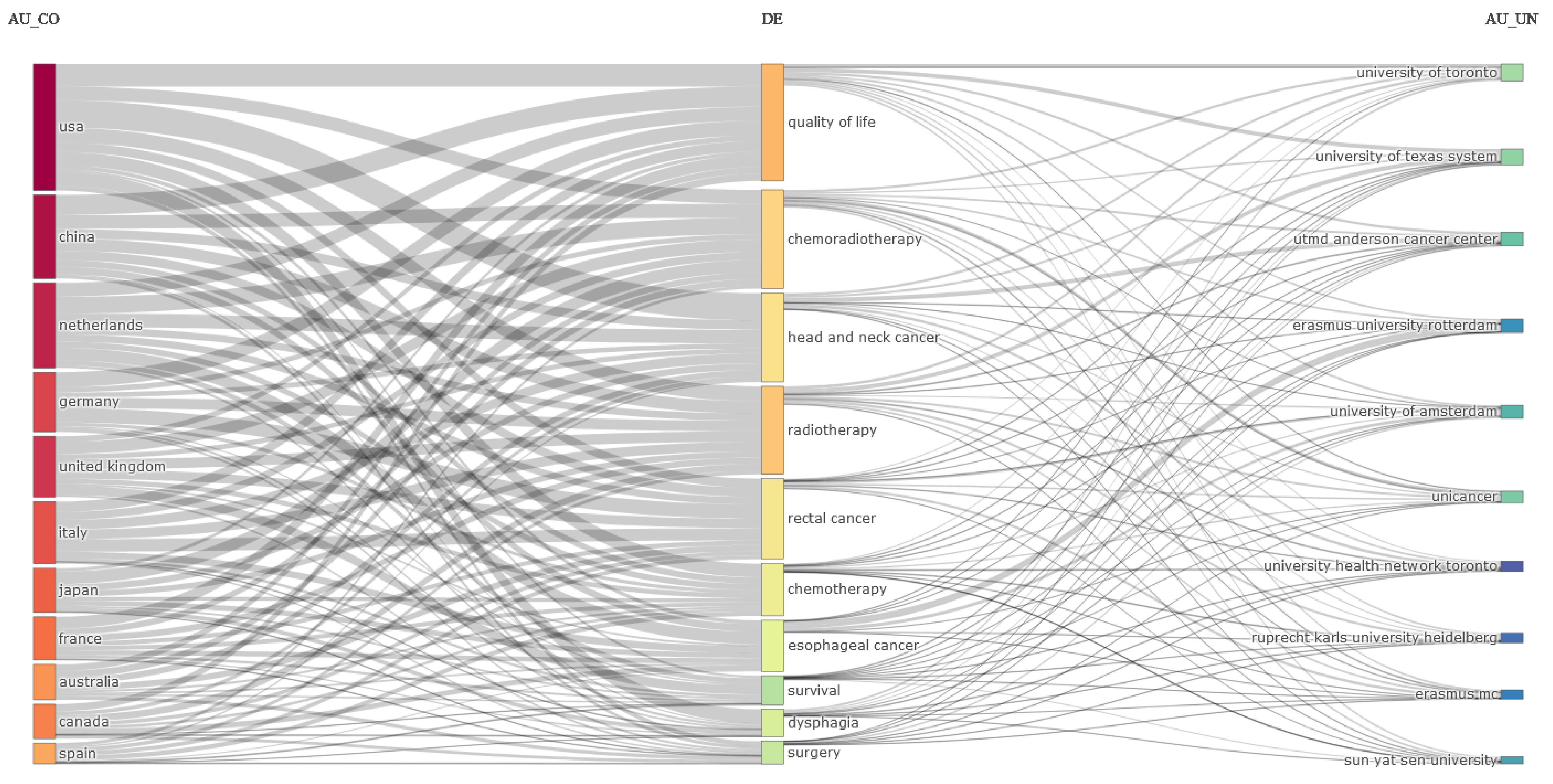

Supplement: Supplementary Figure 4 — Three-field plot of the authors’ keywords analysis (Left field: countries; Middle field: keywords; Right field: institutions). [file Image4.tif]

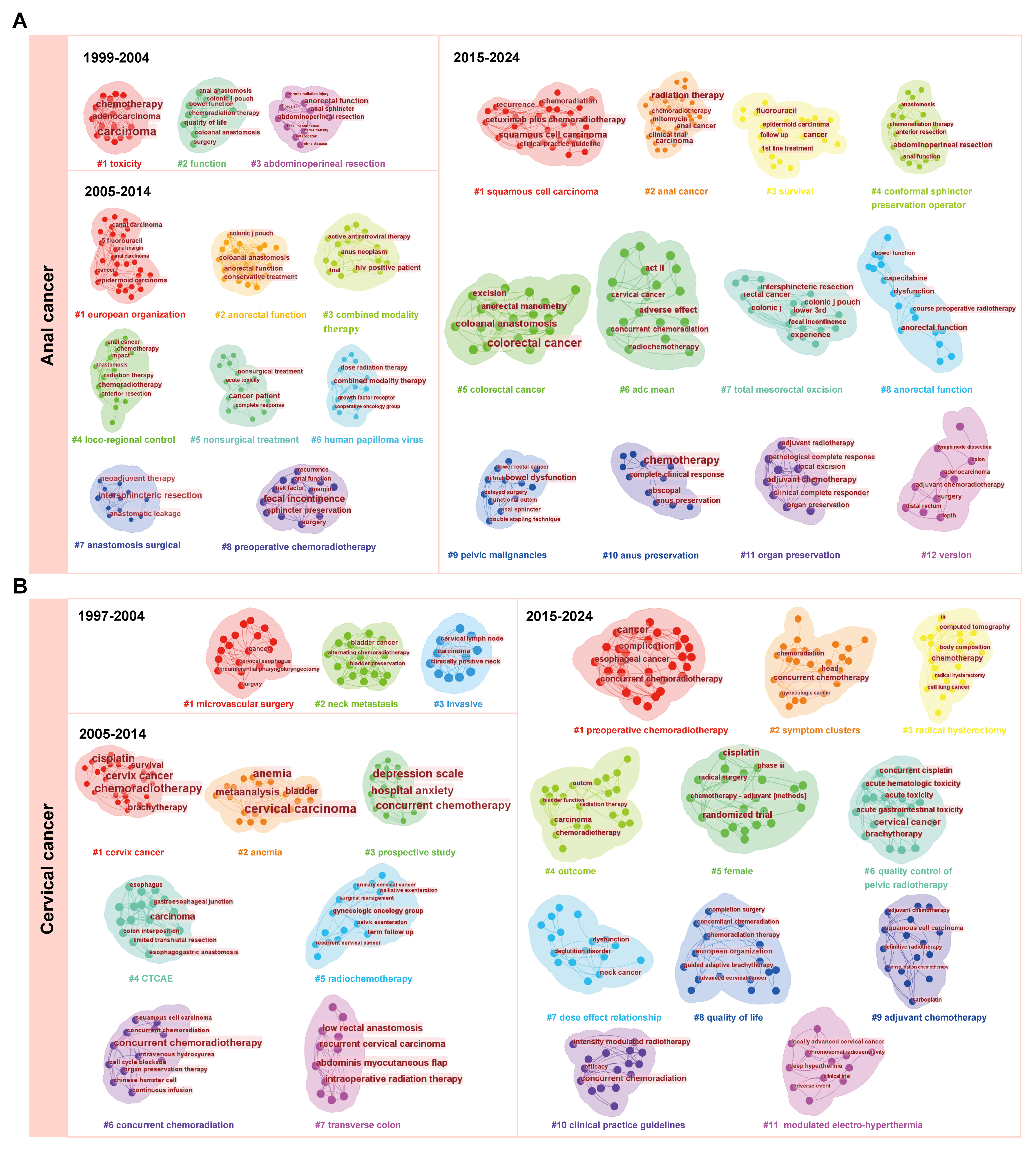

Supplement: Supplementary Figure 5 — Clusters analysis of anal cancer and cervical cancer. (A) The development of the clusters about anal cancer; (B) The development of the clusters about cervical cancer. [file Image5.tif]

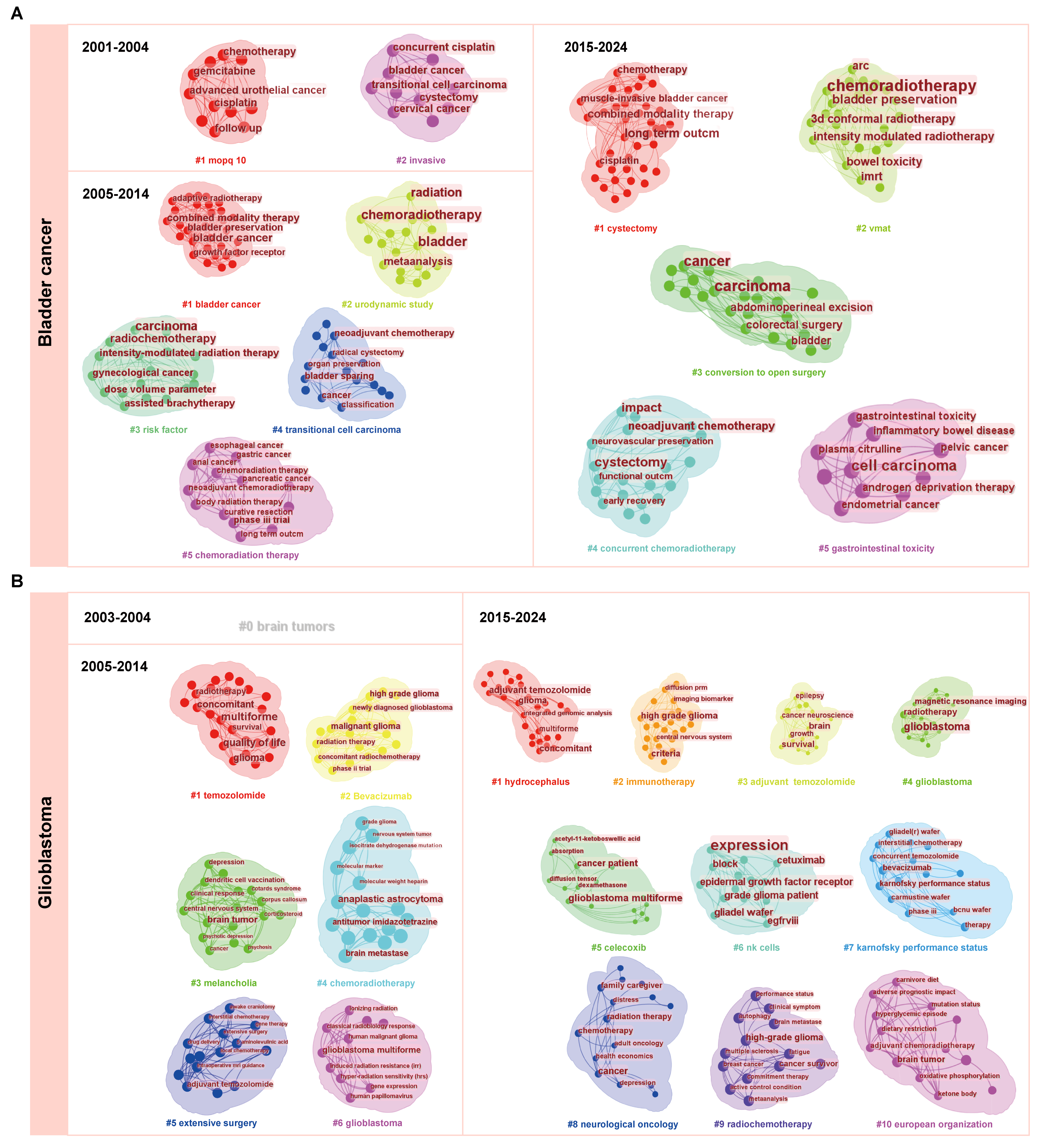

Supplement: Supplementary Figure 6 — Clusters analysis of bladder cancer and glioblastoma. (A) The development of the clusters about bladder cancer; (B) The development of the clusters about glioblastoma. [file Image6.tif]
