# Supplementary material for: Quality of life in patients with pan-cancer undergoing concurrent chemoradiotherapy: a bibliometric analysis (1995-2024)
Source: Front Oncol. 2025 Aug 12;15:1572725. doi: 10.3389/fonc.2025.1572725 (PMC12378759; doi:10.3389/fonc.2025.1572725)
Supplement: Supplementary file 7 [file Table1.docx]

**Table S1. The primary search strategy**

| **Rank** | **Search Strategy** |
| --- | --- |
| **#1** | TS = (Tumor OR Neoplasm OR Tumors OR Neoplasia OR Neoplasias OR Cancer OR Cancers OR Neoplasms OR Head and Neck Neoplasm OR Head and Neck Cancer OR Upper Aerodigestive Tract Neoplasms OR UADT Neoplasm OR UADT Neoplasms OR Upper Aerodigestive Tract Neoplasm OR Head Neoplasms OR Head Neoplasm OR Neck Neoplasms OR Neck Neoplasm OR Head Cancers OR Head Cancer OR Neck Cancers OR Neck Cancer OR Rectal Neoplasms OR Rectal Neoplasm OR Rectum Neoplasms OR Rectum Neoplasm OR Rectal Tumors OR Rectal Tumor OR Rectum Cancers OR Rectal Cancer OR Rectal Cancers OR Rectum Cancer OR Esophageal Neoplasms OR Esophageal Neoplasm OR Esophagus Neoplasm OR Esophagus Neoplasms OR Esophagus Cancers OR Esophageal Cancer OR Esophageal Cancers OR Nasopharyngeal Neoplasms OR Nasopharyngeal Neoplasm OR Nasopharynx Neoplasms OR Nasopharynx Neoplasm OR Nasopharynx Cancers OR Nasopharyngeal Cancer OR Nasopharyngeal Cancers OR Nasopharynx Cancer OR Uterine Cervical Neoplasms OR Uterine Cervical Neoplasm OR Cervical Neoplasms OR Cervical Neoplasm OR Cervix Neoplasm OR Cervix Neoplasms OR Cervical Cancer OR Cervical Cancers OR Uterine Cervical Cancer OR Uterine Cervical Cancers OR Cervix Cancer OR Oropharyngeal Neoplasms OR Oropharyngeal Neoplasm OR Oropharynx Neoplasms OR Oropharynx Neoplasm OR Oropharnyx Cancer OR Oropharnyx Cancers OR Oropharyngeal Cancer OR Oropharyngeal Cancers OR Oropharynx Cancer OR Oropharynx Cancers OR Pancreatic Neoplasms OR Pancreatic Neoplasm OR Pancreas Neoplasms OR Pancreas Neoplasm OR Pancreatic Carcinoma OR Pancreatic Carcinomas OR Pancreas Cancers OR Pancreas Cancer OR Pancreatic Cancer OR Pancreatic Cancers OR Anus Neoplasms OR Anal Neoplasms OR Anal Neoplasm OR Anus Neoplasm OR Anal Cancer OR Anal Cancers OR Anus Cancer OR Anus Cancers OR Stomach Neoplasms OR Stomach Neoplasm OR Gastric Neoplasms OR Gastric Neoplasm OR Stomach Cancers OR Gastric Cancer OR Gastric Cancers OR Stomach Cancer OR Gastric Cancer OR Lung Neoplasms OR Pulmonary Neoplasms OR Lung Neoplasm OR Pulmonary Neoplasm OR Lung Cancer OR Lung Cancers OR Pulmonary Cancer OR Pulmonary Cancers OR Glioblastoma OR Glioblastomas OR Grade IV Astrocytoma OR Grade IV Astrocytomas OR Glioblastoma Multiforme OR Liver Neoplasms OR Liver Neoplasm OR Hepatic Neoplasms OR Hepatic Neoplasm OR Hepatocellular Cancer OR Hepatocellular Cancers OR Hepatic Cancer OR Hepatic Cancers OR Liver Cancer OR Liver Cancers OR Brain Neoplasms OR Brain Neoplasm OR Brain Tumors OR Brain Tumor OR Brain Metastases OR Brain Metastase OR Brain Cancer OR Brain Cancers OR Intracranial Neoplasm OR Intracranial Neoplasms ) |
| **#2** | TS = (quality of life OR HRQoL OR Health related quality of life) |
| **#3** | TS = (Chemoradiotherapies OR Radiochemotherapy OR Radiochemotherapies OR Chemoradiotherapy) |
| **#4** | #1 and #2 and #3 |
