# Supplementary material for: Quality of life in patients with pan-cancer undergoing concurrent chemoradiotherapy: a bibliometric analysis (1995-2024)
Source: Front Oncol. 2025 Aug 12;15:1572725. doi: 10.3389/fonc.2025.1572725 (PMC12378759; doi:10.3389/fonc.2025.1572725)
Supplement: Supplementary file 8 [file Table2.docx]

**Table S2. Six cancer types with search strategy**

| **Keyword** | **Search Strategy** | **Article Numbers** |
| --- | --- | --- |
| **Head and neck cancer** | (TS = (Head and Neck Neoplasm OR Cancer of Head and Neck OR Head and Neck Cancer OR Cancer of the Head and Neck OR Upper Aerodigestive Tract Neoplasms OR UADT Neoplasm OR UADT Neoplasms OR Upper Aerodigestive Tract Neoplasm OR Head Neoplasms OR Head Neoplasm OR Neck Neoplasms ORNeck Neoplasm OR Cancer of Head OR Head Cancers OR Head Cancer OR Cancer of the Head OR Cancer of Neck OR Neck Cancers OR Neck Cancer OR Cancer of the Neck)) AND (TS = (quality of life OR HRQoL OR Health related quality of life)) AND (TS = (Chemoradiotherapies OR Radiochemotherapy OR Radiochemotherapies OR Chemoradiotherapy)) | 1014 |
| **Rectal cancer** | (TS = (Rectal Neoplasms OR Rectal Neoplasm OR Rectum Neoplasms OR Rectum Neoplasm OR Rectal Tumors OR Rectal Tumor OR Cancer of Rectum OR Rectum Cancers OR Rectal Cancer OR Rectal Cancers OR Rectum Cancer OR Cancer of the Rectum)) AND (TS = (quality of life OR HRQoL OR Health related quality of life)) AND (TS = (Chemoradiotherapies OR Radiochemotherapy OR Radiochemotherapies OR Chemoradiotherapy)) | 611 |
| **Anal cancer** | (TS = (Anus Neoplasms OR Anal Neoplasms OR Anal Neoplasm OR Anus Neoplasm OR Anal Cancer OR Anal Cancers OR Anus Cancer OR Anus Cancers) AND (TS = (quality of life OR HRQoL OR Health related quality of life)) AND (TS = (Chemoradiotherapies OR Radiochemotherapy OR Radiochemotherapies OR Chemoradiotherapy)) | 186 |
| **Cervical cancer** | (TS = (Uterine Cervical Neoplasms OR Uterine Cervical Neoplasm OR Cervical Neoplasms OR Cervical Neoplasm OR Cervix Neoplasm OR Cervix Neoplasms OR Cervical Cancer OR Cervical Cancers OR Uterine Cervical Cancer OR Uterine Cervical Cancers OR Cancer of Cervix OR Cervix Cancer)) AND (TS = (quality of life OR HRQoL OR Health related quality of life)) AND (TS = (Chemoradiotherapies OR Radiochemotherapy OR Radiochemotherapies OR Chemoradiotherapy)) | 195 |
| **Bladder cancer** | (TS = (Bladder Tumors OR Bladder Tumor OR Bladder Neoplasms OR Bladder Neoplasm OR Bladder Cancer OR Bladder Cancers)) AND (TS = (quality of life OR HRQoL OR Health related quality of life)) AND (TS = (Chemoradiotherapies OR Radiochemotherapy OR Radiochemotherapies OR Chemoradiotherapy)) | 870 |
| **Glioblastoma** | (TS = (Glioblastoma OR Glioblastomas OR Grade IV Astrocytoma OR Grade IV Astrocytomas OR Glioblastoma Multiforme)) AND (TS = (quality of life OR HRQoL OR Health related quality of life)) AND (TS = (Chemoradiotherapies OR Radiochemotherapy OR Radiochemotherapies OR Chemoradiotherapy)) | 72 |
