# Supplementary material for: Quality of life in patients with pan-cancer undergoing concurrent chemoradiotherapy: a bibliometric analysis (1995-2024)
Source: Front Oncol. 2025 Aug 12;15:1572725. doi: 10.3389/fonc.2025.1572725 (PMC12378759; doi:10.3389/fonc.2025.1572725)
Supplement: Supplementary file 9 [file Table3.docx]

**Table S3. The top 10 authors in publication and their research contents**

| **Rank** | **Authors** | **Articles** | **Institution** | **Country** | **H-index** | **Research contents** |
| --- | --- | --- | --- | --- | --- | --- |
| 1 | Henegouwen, Mark I. van Berge | 27 | University of Amsterdam | NETHERLANDS | 16 | Surgery; Gastroenterology & Hepatology; Oncology; Respiratory System; Radiology, Nuclear Medicine & Medical Imaging |
| 2 | Bas Wijnhoven | 25 | Erasmus University Medical Center | NETHERLANDS | 14 | Surgery; Oncology; Gastroenterology & Hepatology Pathology; Respiratory System |
| 3 | Everett Vokes. | 21 | University of Chicago | UNITED STATES | 16 | Oncology; Respiratory System; Radiology, Nuclear Medicine & Medical Imaging; Pharmacology & Pharmacy Surgery |
| 4 | Lagarde, Sjoerd M. | 20 | Erasmus University Rotterdam | NETHERLANDS | 13 | Surgery; Oncology; Gastroenterology & Hepatology; Respiratory System; Cardiovascular System & Cardiology |
| 5 | van Hillegersberg, Richard. | 20 | Utrecht University Medical Center | NETHERLANDS | 13 | Surgery; Oncology; Gastroenterology & Hepatology; Radiology, Nuclear Medicine & Medical Imaging; Respiratory System |
| 6 | Hutcheson, Katherine A. | 19 | UTMD Anderson Cancer Center | UNITED STATES | 11 | Oncology; Radiology, Nuclear Medicine & Medical Imaging; Otorhinolaryngology; Surgery; Research & Experimental Medicine |
| 7 | van Lanschot, J. Jan B. | 19 | Erasmus MC Cancer Institute | NETHERLANDS | 15 | Surgery; Gastroenterology & Hepatology; Oncology; Cardiovascular System & Cardiology; Respiratory System |
| 8 | Nieuwenhuijzen, Grard A. P. | 18 | Catharina Hospital | NETHERLANDS | 9 | Oncology; Surgery Gastroenterology & Hepatology; Radiology, Nuclear Medicine & Medical Imaging; General & Internal Medicine |
| 9 | Reynolds, John V. | 18 | Trinity St James Cancer Inst | IRELAND | 12 | Surgery; Oncology; Gastroenterology & Hepatology; General & Internal Medicine Immunology |
| 10 | van Laarhoven, Hanneke W. M. | 18 | Amsterdam UMC Locat Univ Amsterdam | NETHERLANDS | 10 | Oncology; Radiology, Nuclear Medicine & Medical Imaging; Health Care Sciences & Services; Surgery; Gastroenterology & Hepatology |
