# Supplementary material for: Quality of life in patients with pan-cancer undergoing concurrent chemoradiotherapy: a bibliometric analysis (1995-2024)
Source: Front Oncol. 2025 Aug 12;15:1572725. doi: 10.3389/fonc.2025.1572725 (PMC12378759; doi:10.3389/fonc.2025.1572725)
Supplement: Supplementary file 10 [file Table4.docx]

**Table S4. The top 10 articles with the most local citations**

| **Rank** | **Title** | **Author** | **Year** | **Local Citations** | **Type of research** |
| --- | --- | --- | --- | --- | --- |
| 1 | Preoperative radiotherapy versus selective postoperative chemoradiotherapy in patients with rectal cancer (MRC CR07 and NCIC-CTG C016): a multicentre, randomised trial | SEBAG-MONTEFIORE D | 2009 | 86 | Randomized controlled trial |
| 2 | Quality of Life and Performance in Advanced Head and Neck Cancer Patients on Concomitant Chemoradiotherapy: A Prospective Examination | LIST MA | 1999 | 75 | Prospective research |
| 3 | Intensity-Modulated Chemoradiotherapy Aiming to Reduce Dysphagia in Patients with Oropharyngeal Cancer: Clinical and Functional Results | FENG FY | 2010 | 53 | Prospective research |
| 4 | Factors Associated with Long-Term Dysphagia After Definitive Radiotherapy for Locally Advanced Head-and-Neck Cancer | CAUDELL JJ | 2009 | 51 | Prospective research |
| 5 | Prevention and Treatment of Dysphagia and Aspiration after Chemoradiation for Head and Neck Cancer | ROSENTHAL DI | 2006 | 48 | Review |
| 6 | Effect of Neoadjuvant Chemoradiotherapy on Health-Related Quality of Life in Esophageal or Junctional Cancer: Results from the Randomized CROSS Trial | NOORDMAN BJ | 2018 | 46 | Randomized controlled trial |
| 7 | Quality of life, anorectal and sexual functions after preoperative radiotherapy for rectal cancer: Report of a randomised trial | PIETRZAK L | 2007 | 44 | Randomized controlled trial |
| 8 | Eat and Exercise During Radiotherapy or Chemoradiotherapy for Pharyngeal CancersUse It or Lose It | HUTCHESON KA | 2013 | 44 | Retrospective observational study |
| 9 | Minimally invasive versus open oesophagectomy for patients with oesophageal cancer: a multicentre, open-label, randomised controlled trial | BIERE SSAY | 2012 | 43 | Randomized controlled trial |
| 10 | Radiotherapy plus cetuximab or cisplatin in human papillomavirus-positive oropharyngeal cancer (NRG Oncology RTOG 1016): a randomised, multicentre, non-inferiority trial | GILLISON ML | 2019 | 43 | Randomized controlled trial |
