# Supplementary material for: Quality of life in patients with pan-cancer undergoing concurrent chemoradiotherapy: a bibliometric analysis (1995-2024)
Source: Front Oncol. 2025 Aug 12;15:1572725. doi: 10.3389/fonc.2025.1572725 (PMC12378759; doi:10.3389/fonc.2025.1572725)
Supplement: Supplementary file 11 [file Table5.docx]

**Table S5. The top 10 articles with the most global citations**

| **Rank** | **Title** | **Author** | **Year** | **Global Citations** | **Type of research** |
| --- | --- | --- | --- | --- | --- |
| 1 | Bevacizumab plus Radiotherapy–Temozolomide for Newly Diagnosed Glioblastoma | CHINOT OL | 2014 | 1736 | Randomized controlled trial |
| 2 | Effect of Tumor-Treating Fields Plus Maintenance Temozolomide vs Maintenance Temozolomide Alone on Survival in Patients with Glioblastoma: A Randomized Clinical Trial | STUPP R | 2017 | 1394 | Randomized controlled trial |
| 3 | Head and neck squamous cell carcinoma | JOHNSON DE | 2020 | 1267 | Review |
| 4 | Minimally invasive versus open oesophagectomy for patients with oesophageal cancer: a multicentre, open-label, randomised controlled trial | BIERE SSAY | 2012 | 1183 | Randomized controlled trial |
| 5 | Preoperative radiotherapy versus selective postoperative chemoradiotherapy in patients with rectal cancer (MRC CR07 and NCIC-CTG C016): a multicentre, randomised trial | SEBAG-MONTEFIORE D | 2009 | 1091 | Randomized controlled trial |
| 6 | Adjuvant Chemotherapy with Fluorouracil Plus Folinic Acid vs Gemcitabine Following Pancreatic Cancer Resection: A Randomized Controlled Trial | NEOPTOLEMOS JP | 2010 | 1027 | Randomized controlled trial |
| 7 | Pancreatic cancer: A review of clinical diagnosis, epidemiology, treatment and outcomes | MCGUIGAN A | 2018 | 1021 | Review |
| 8 | Cholangiocarcinoma | KHAN SA | 2005 | 960 | Review |
| 9 | Maintenance Therapy with Tumor-Treating Fields Plus Temozolomide vs Temozolomide Alone for Glioblastoma: A Randomized Clinical Trial | STUPP R | 2015 | 841 | Randomized controlled trial |
| 10 | Radiotherapy plus cetuximab or cisplatin in human papillomavirus-positive oropharyngeal cancer (NRG Oncology RTOG 1016): a randomised, multicentre, non-inferiority trial | GILLISON ML | 2019 | 786 | Randomized controlled trial |
