# Supplementary material for: Quality of life in patients with pan-cancer undergoing concurrent chemoradiotherapy: a bibliometric analysis (1995-2024)
Source: Front Oncol. 2025 Aug 12;15:1572725. doi: 10.3389/fonc.2025.1572725 (PMC12378759; doi:10.3389/fonc.2025.1572725)
Supplement: Supplementary file 12 [file Table6.docx]

**Table S6. Frequency table of the top 50 authors’ keywords**

| **Rank** | **Keywords** | **Frequency** |  | **Rank** | **Keywords** | **Frequency** |
| --- | --- | --- | --- | --- | --- | --- |
| 1 | quality of life | 407 |  | 26 | oropharyngeal cancer | 39 |
| 2 | radiotherapy | 378 |  | 27 | pancreatic cancer | 39 |
| 3 | chemoradiotherapy | 356 |  | 28 | oral mucositis | 37 |
| 4 | head and neck cancer | 312 |  | 29 | swallowing | 35 |
| 5 | rectal cancer | 283 |  | 30 | treatment | 35 |
| 6 | chemotherapy | 191 |  | 31 | anal cancer | 34 |
| 7 | esophageal cancer | 127 |  | 32 | gastric cancer | 34 |
| 8 | dysphagia | 109 |  | 33 | immunotherapy | 34 |
| 9 | surgery | 107 |  | 34 | meta-analysis | 34 |
| 10 | survival | 84 |  | 35 | mucositis | 34 |
| 11 | chemoradiation | 83 |  | 36 | head and neck neoplasms | 33 |
| 12 | nasopharyngeal carcinoma | 77 |  | 37 | cisplatin | 32 |
| 13 | cancer | 76 |  | 38 | glioblastoma | 32 |
| 14 | radiochemotherapy | 65 |  | 39 | prognosis | 32 |
| 15 | organ preservation | 64 |  | 40 | lung cancer | 31 |
| 16 | cervical cancer | 62 |  | 41 | nutrition | 30 |
| 17 | toxicity | 62 |  | 42 | induction chemotherapy | 29 |
| 18 | concurrent chemoradiotherapy | 57 |  | 43 | patient-reported outcomes | 29 |
| 19 | neoadjuvant chemoradiotherapy | 54 |  | 44 | systematic review | 29 |
| 20 | neoadjuvant therapy | 54 |  | 45 | elderly | 28 |
| 21 | radiation therapy | 54 |  | 46 | imrt | 28 |
| 22 | esophagectomy | 53 |  | 47 | intensity-modulated radiotherapy | 28 |
| 23 | squamous cell carcinoma | 46 |  | 48 | non-small cell lung cancer | 28 |
| 24 | health-related quality of life | 45 |  | 49 | deglutition | 27 |
| 25 | radiation | 44 |  | 50 | exercise | 27 |
