# Supplementary material for: Quality of life in patients with pan-cancer undergoing concurrent chemoradiotherapy: a bibliometric analysis (1995-2024)
Source: Front Oncol. 2025 Aug 12;15:1572725. doi: 10.3389/fonc.2025.1572725 (PMC12378759; doi:10.3389/fonc.2025.1572725)
Supplement: Supplementary file 13 [file Table7.docx]

**Table S7. Sociodemographic and clinical characteristics of the patients (*N* = 117)**

| **Variable** | **Category** | **n (%)** |
| --- | --- | --- |
| Age (years) | 26-30 | 2(1.71%) |
|  | 31-40 | 5(4.27%) |
|  | 41-50 | 15(12.8%) |
|  | 51-70 | 94(80.3%) |
|  | ＞70 | 1(0.9%) |
| Education | ≤ Junior high school | 94(80.3%) |
|  | High school | 15(12.8%) |
|  | ≥College | 8(6.8%) |
| Marital status | Never married | 2(1.7%) |
|  | Married | 3(2.6%) |
|  | Divorced/widowed | 112(95.7%) |
| Monthly income per capita (RMB) | <3,000 | 40(34.2%) |
|  | 3,000–5,000 | 47(40.2%) |
|  | >5,000 | 30(25.6%) |
| Histologic Types | Squamous Cell Carcinoma | 106(90.6%) |
|  | Adenocarcinoma | 8(6.8%) |
|  | Other types | 3(2.6%) |
| Stage | I, II | 50(42.7%) |
|  | III, IV | 67(57.3%) |
