# Supplementary material for: Quality of life in patients with pan-cancer undergoing concurrent chemoradiotherapy: a bibliometric analysis (1995-2024)
Source: Front Oncol. 2025 Aug 12;15:1572725. doi: 10.3389/fonc.2025.1572725 (PMC12378759; doi:10.3389/fonc.2025.1572725)
Supplement: Supplementary file 14 [file Table8.docx]

**Table S8. The severity and prevalence of symptoms and clusters**

| **Rank** | **Symotom** | **Prevalence (%)** | **Clusters** | **Severity mean ± SD** |
| --- | --- | --- | --- | --- |
| 1 | Xerostomia | 41.9 | 1 | 0.79±1.09 |
| 2 | Numbness/Tingling | 25.6 | 1 | 0.56±1.09 |
| 3 | Burping | 23.1 | 1 | 0.46±0.95 |
| 4 | Diarrhea | 65.8 | 1 | 1.59±1.38 |
| 5 | Abdominal pain | 27.4 | 1 | 0.51±0.91 |
| 6 | Hypomnesia | 23.9 | 1 | 0.52±1.03 |
| 7 | Myalgia | 21.4 | 1 | 0.47±0.99 |
| 8 | Vaginal bleeding | 31.6 | 1 | 0.49±0.78 |
| 9 | Dizziness | 24.8 | 2 | 0.44±0.86 |
| 10 | Bloating | 21.4 | 2 | 0.44±0.96 |
| 11 | Constipation | 21.4 | 2 | 0.50±1.04 |
| 12 | Alopecia | 37.6 | 2 | 1.10±1.56 |
| 13 | Frequent urination | 37.6 | 2 | 0.71±1.09 |
| 14 | Painful urination | 47 | 2 | 0.86±1.12 |
| 15 | Hot flashes and night sweats | 46.2 | 2 | 1.05±1.25 |
| 16 | Insomnia | 34.2 | 3 | 0.73±1.11 |
| 17 | Fatigue | 53 | 3 | 1.06±1.13 |
| 18 | Decreased libido | 23.1 | 3 | 0.53±1.08 |
| 19 | Altered taste | 59 | 3 | 1.45±1.38 |
| 20 | Decreased appetite | 79.5 | 3 | 2.02±1.25 |
| 21 | Nausea and vomiting | 58.1 | 3 | 1.44±1.4 |
| 22 | Dry skin | 29.9 | 3 | 0.51±0.88 |
| 23 | Hyperpigmentation | 42.7 | 3 | 0.69±0.95 |
